# Structural analysis of the 2-oxoglutarate binding site of the circadian rhythm linked oxygenase JMJD5

**DOI:** 10.1038/s41598-022-24154-0

**Published:** 2022-11-30

**Authors:** Md. Saiful Islam, Marios Markoulides, Rasheduzzaman Chowdhury, Christopher J. Schofield

**Affiliations:** Chemistry Research Laboratory, Department of Chemistry and the Ineos Oxford Institute for Antimicrobial Research, University of Oxford, 12 Mansfield Road, Oxford, OX1 3TA UK

**Keywords:** Metabolomics, Biochemistry, Cell biology

## Abstract

JmjC (Jumonji-C) domain-containing 5 (JMJD5) plays important roles in circadian regulation in plants and humans and is involved in embryonic development and cell proliferation. JMJD5 is a 2-oxoglutarate (2OG) and Fe(II) dependent oxygenase of the JmjC subfamily, which includes histone N^ε^-methyl lysine-demethylases (KDMs) and hydroxylases catalysing formation of stable alcohol products. JMJD5 is reported to have KDM activity, but has been shown to catalyse C-3 hydroxylation of arginine residues in sequences from human regulator of chromosome condensation domain-containing protein 1 (RCCD1) and ribosomal protein S6 (RPS6) in vitro. We report crystallographic analyses of human JMJD5 complexed with 2OG analogues, including the widely used hypoxia mimic pyridine-2,4-dicarboxylate, both D- and L-enantiomers of the oncometabolite 2-hydroxyglutarate, and a cyclic *N*-hydroxyimide. The results support the assignment of JMJD5 as a protein hydroxylase and reveal JMJD5 has an unusually compact 2OG binding pocket suitable for exploitation in development of selective inhibitors. They will be useful in the development of chemical probes to investigate the physiologically relevant roles of JMJD5 in circadian rhythm and development and explore its potential as a medicinal chemistry target.

## Introduction

2-Oxoglutarate (2OG)- and Fe(II)-dependent oxygenases (2OG-oxygenases) play key roles in human biology, including in collagen biosynthesis, lipid metabolism, nucleic acid repair/ modification, the regulation of protein stability/ biosynthesis, and in the hypoxic response^1–4^. They are also clinically validated therapeutic targets^5^ as shown by the development of inhibitors of the hypoxia inducible factor prolyl hydroxylases (PHDs or EGLNs) for the treatment of anaemia in kidney disease.^6–8^ 2OG dependent histone N^ε^-methyl lysine-demethylases (KDMs) are also being targeted for the treatment of cancer^9^. Meldonium (Mildronate, 2-(2-carboxylato-ethyl)-1,1,1-trimethylhydrazinium) is a 2OG-oxygenase inhibitor that is approved in some countries for treatment of heart disease and which has been used by athletes to improve performance^10–12^. Meldonium is a metabolic regulator, which inhibits γ-butyrobetaine hydroxylase (BBOX), a 2OG-oxygenase that catalyses the final step in carnitine biosynthesis^13,14^.

With the exception of Meldonium, most, it not all, clinically used/ evaluated PHD inhibitors compete with 2OG for coordination with the single active site Fe(II), which is present in all characterised 2OG oxygenases^15^. The development of such PHD inhibitors was important in part because it demonstrated that such a mechanism of action can apparently be safe, despite the use of 2OG by 60–70 human 2OG oxygenases and many other enzymes, including tricarboxylic acid (TCA) cycle and related metabolic enzymes. Nonetheless, there is evidence that the selectivity of the current PHD inhibitors can be improved^8^ and the development of other 2OG oxygenases as drug targets has been limited by selectivity concerns.

The collagen prolyl-4-hydroxylases (CP4Hs) were the first human 2OG-oxygenases to be targeted for inhibition, e.g. for treatment of fibrotic diseases by 2OG analogues / competitors, such as pyridine-2,4-dicarboxylic acid (2,4-PDCA), N-oxalylglycine (NOG), and their derivatives, as well as other bidentate Fe(II) chelators^16–18^. However, efforts to develop therapeutically useful CP4H inhibitors have to date been unsuccessful, likely in part due to off-target effects, including non-specific inhibition of other 2OG-oxygenases, probably including the PHDs. Despite selectivity issues, 2,4-PDCA and, in particular, NOG have been and continue to be widely used as hypoxia mimics in their prodrug forms, likely in large part via inhibition of the PHDs. Selectivity concerns have also hindered drug development work on other human 2OG oxygenases, including the JmjC subfamily enzymes^19–25^, which includes histone N^ε^-methyl lysine-demethylases (KDMs) and N^ε^-methyl arginine-demethylases, as well as hydroxylases forming stable alcohol products^3,5,9^. Detailed structural knowledge of differences in 2OG binding modes manifested by 2OG-oxygenases can be used to develop selective inhibitors, as shown by work with the JmjC hydroxylase factor inhibitor HIF (FIH)^26^.

**Figure 1:**
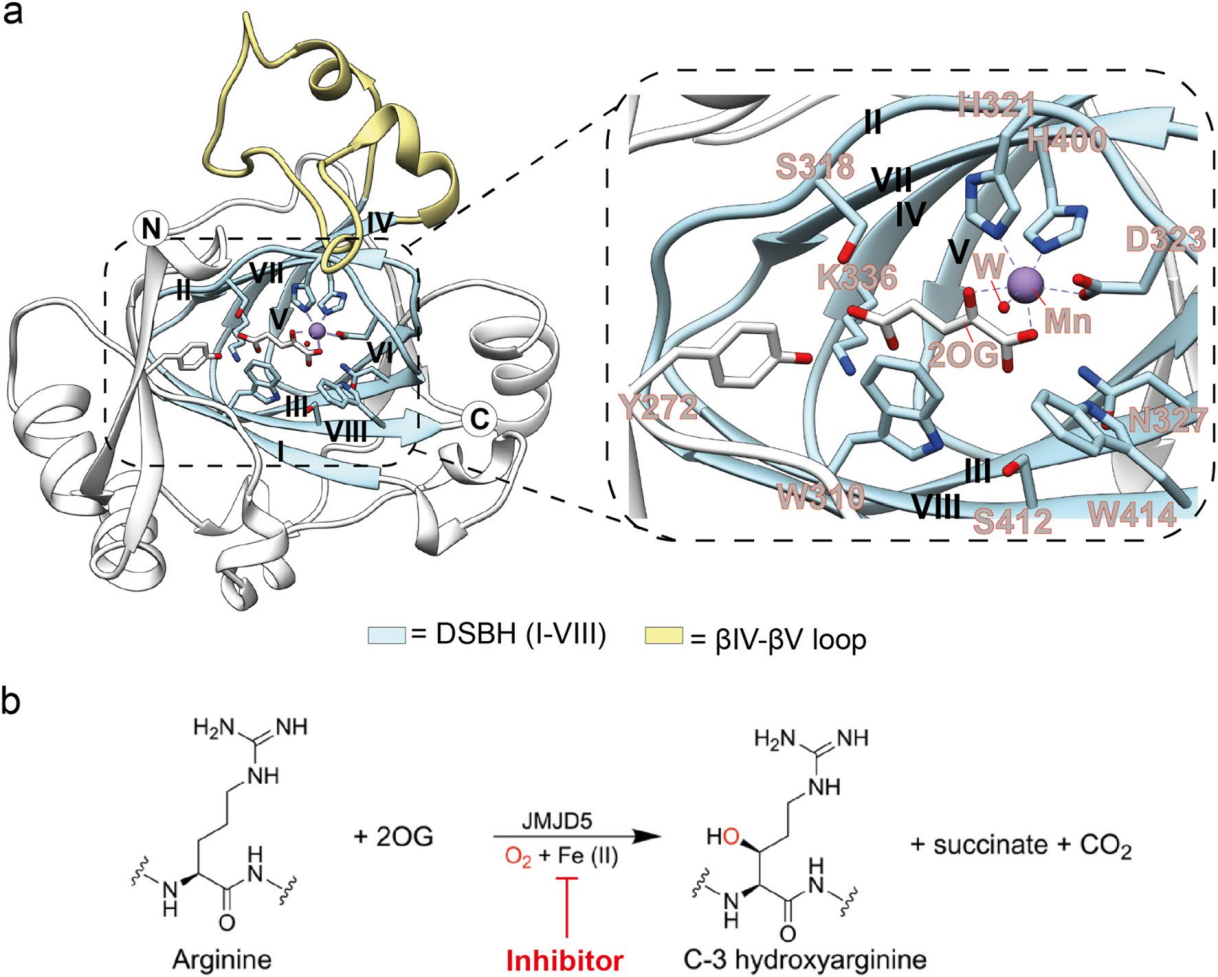
JMJD5 catalyses C-3 arginine-residue hydroxylation. (**a**) View derived from a crystal structure of JMJD5^∆1–181^ in complex with Mn (substituting for catalytically active Fe) and 2OG (PDB: 6F4N) showing the core distorted double stranded β-helix (DSBH) fold, the eight β-strands of which are labelled I-VIII; the loop linking βIV and βV of the DSBH which is likely involved in substrate binding is shown. The inset shows key active site residues (H321, H400, and D323), Mn (replacing Fe), and 2OG and 2OG-binding residues, including K336, Y272, and W310. (**b**) Arginine C-3 hydroxylation as catalysed by JMJD5 is inhibited by 2OG mimetics.

Although the JmjC protein JMJD5 has been reported to have H3K9Me_n_ KDM activity, it has also been shown to catalyse stereoselective C-3 hydroxylation of arginine residues in sequences from human regulator of chromosome condensation domain-containing protein (RCCD1) and ribosomal protein S6 (RPS6) (Fig. 1)^27,28^. JMJD5 has also been reported to have histone endopeptidase activity^29^ and studies with animals and cells have shown that JMJD5 is involved in embryonic development^30^. Strikingly, evidence has been presented that JMJD5 has a role in the circadian rhythm in both plants and humans^31^, as well as in the regulation of RNA polymerase II^32^. Compared to the wildtype JMJD5s, the *Arabidopsis thaliana JMJD5* mutant seedlings and mammalian cell cultures deficient in *JMJD5* have similarly faster rates of circadian oscillations; notably the evolutionary distant JMJD5 orthologs are sufficiently conserved to be interchangeable between plant and mammalian cells^31^. However, the relationships between the physiological role of JMJD5 in circadian regulation and its catalytic / biochemical roles in cells are not understood.

Here we report structural and biochemical studies investigating inhibitor-binding in the 2OG binding pocket of human JMJD5. The results reveal a distinct, unusually compact and druggable 2OG binding pocket, and identify scaffolds, including a cyclic *N*-hydroxyimide, that are suitable for development of selective JMJD5 inhibitors. The results also reveal the potential for inhibition of JMJD5 by the oncometabolite 2-hydroxyglurate, which is upregulated in cancer cells due to mutations to isocitrate dehydrogenases 1 and 2 (IDH 1/2)^33,34^.

## Results

### Inhibition of JMJD5

To explore inhibition involving orthosteric 2OG ligand binding in the JMJD5 2OG pocket, we initially screened a focused set of compounds comprising relatively close 2OG mimetics, tricarboxylic acid (TCA) cycle intermediates, hypoxia inducible factor (HIF) prolyl hydroxylase (PHD) inhibitors, and JmjC histone demethylase (KDM) inhibitors (Supplementary Fig. S1). The compounds were initially screened at three fixed concentrations (0.1, 1 and 10 mM) (Supplementary Fig. S2), with selected compounds being chosen for IC_50_ determinations. The assays employed monitoring of full length JMJD5 (10 µM) catalysed hydroxylation of RpS6_129-144_ (VPRRLGPKASRIRKL) in the presence of ferrous iron, 2OG, and *L*-ascorbate, as measured by matrix assisted laser desorption ionisation (MALDI) mass spectrometry analysis (Fig. 1)^28^. Compounds showing ≥ 50% inhibition at 0.1 mM concentration (Supplementary Fig. S2) were then selected for subsequent IC_50_ determinations using varied inhibitor concentrations (0.05–10 mM) (See Methods for details).

None of the TCA cycle intermediates and related compounds were potent JMJD5 inhibitors; notably, however, *l*-2-hydroxyglutarate (*l*-2HG) was more potent than its pro-oncogenic enantiomer *d*-2-hydroxyglutarate (*d*-2HG)^35^. The combined inhibition results reveal that, at least for the tested compounds in some cases, relatively small inhibitors are more potent against JMJD5 compared to inhibition of structurally related JmjC-oxygenases. Thus, *N*-oxalylglycine (NOG) itself is a more potent inhibitor than either *d*- or *l*- *N*-oxalylalanine, pyridine-2,4-dicarboxylate (2,4 -PDCA) is more potent than bipyridinyl-2,4-dicarboxylate (2,4-BPDCA), and the PHD inhibitor Vadadustat is more potent than FG-4592, a clinically used PHD inhibitor (Table 1, Supplementary Figs S3–S6). An exception to the apparent preference for relatively small inhibitors with the tested compounds is the clinically approved PHD inhibitor Daprodustat (GSK1278863)^8,36^, which contains two cyclohexyl rings and a glycine sidechain, which is a relatively potent JMJD5 inhibitor under the tested conditions (Table 1, Supplementary Fig. S5). Furthermore, the functionalised 8-hydroxyisoquinoline inhibitor ML-324 (a JmjC KDM inhibitor)^37^ was markedly more potent than the simpler 5-carboxy-8-hydroxyquinoline (IOX1), a relatively broad spectrum 2OG oxygenase inhibitor that has been shown to induce iron movement at the active site of some JmjC KDMs ^38^. Given that there are extensive reports of hydroxamic acids as 2OG oxygenase and, in particular, JmjC KDM inhibitors (e.g. the clinically used histone dacetylase inhibitor Vorinostat/Suberoylanilide hydroxamic acid, SAHA)^39^, the observation that cyclic *N*-hydroxyimide **4** is a relatively potent JMJD5 inhibitor is interesting.

**Table 1:** IC_50_ values for JMJD5^FL^ inhibitors that likely bind in the 2OG cosubstrate pocket. A full-length JMJD5 construct (aa 1–416, JMJD5^FL^) was used in the inhibition assays. *IC*_*50*_ values were determined using MALDI-TOF-based hydroxylation assays with 10 µM JMJD5^FL^, 100 µM Fe(II), 400 µM ascorbate, 50 µM 2OG and 100 µM substrate RpS6_129-144_ (VPRRLGPKRASRIRKL) in the presence of varied concentrations of inhibitors (0–10 mM). Values represent mean (n = 3) ± SD.

	*IC*_*50*_ (µM)
**TCA cycle intermediates**	
Citrate^5^	820 ± 1.8
Fumarate^5^	1161 ± .1.4
d-2HG^5^	1268 ± 1.5
Isocitrate^5^	1294 ± 2
l-2HG^5^	340 ± 1.3
Malate^5^	1604 ± 1.4
Oxaloacetate^5^	85 ± 1.4
Pyruvate^5^	500 ± 1.6
Succinate^5^	840 ± 1.4
**2OG mimetics**	
NOG^5^	6 ± 1.4
2.4-PDCA^5^	12 ± 1.4
2.4-BPDCA^5^	66 ± 1.5
**4**^5^	53 ± 1.4
IOX1^38^	96 ± 1.7
**PHD2 inhibitors**	
GSK1278863^36^	9 ± 1.2
FG4592^85^	239 ± 1.9
Vadadustat^86^	54 ± 1.5
AKB-6899^87^	288 ± 1.5
**KDM inhibitors**	
GSK-J1^19^	67 ± 1.5
KDM5-C49^20^	621 ± 1.4
ML-324^37^	15 ± 1.4

### JMJD5 inhibitor complex structures

**Figure 2:**
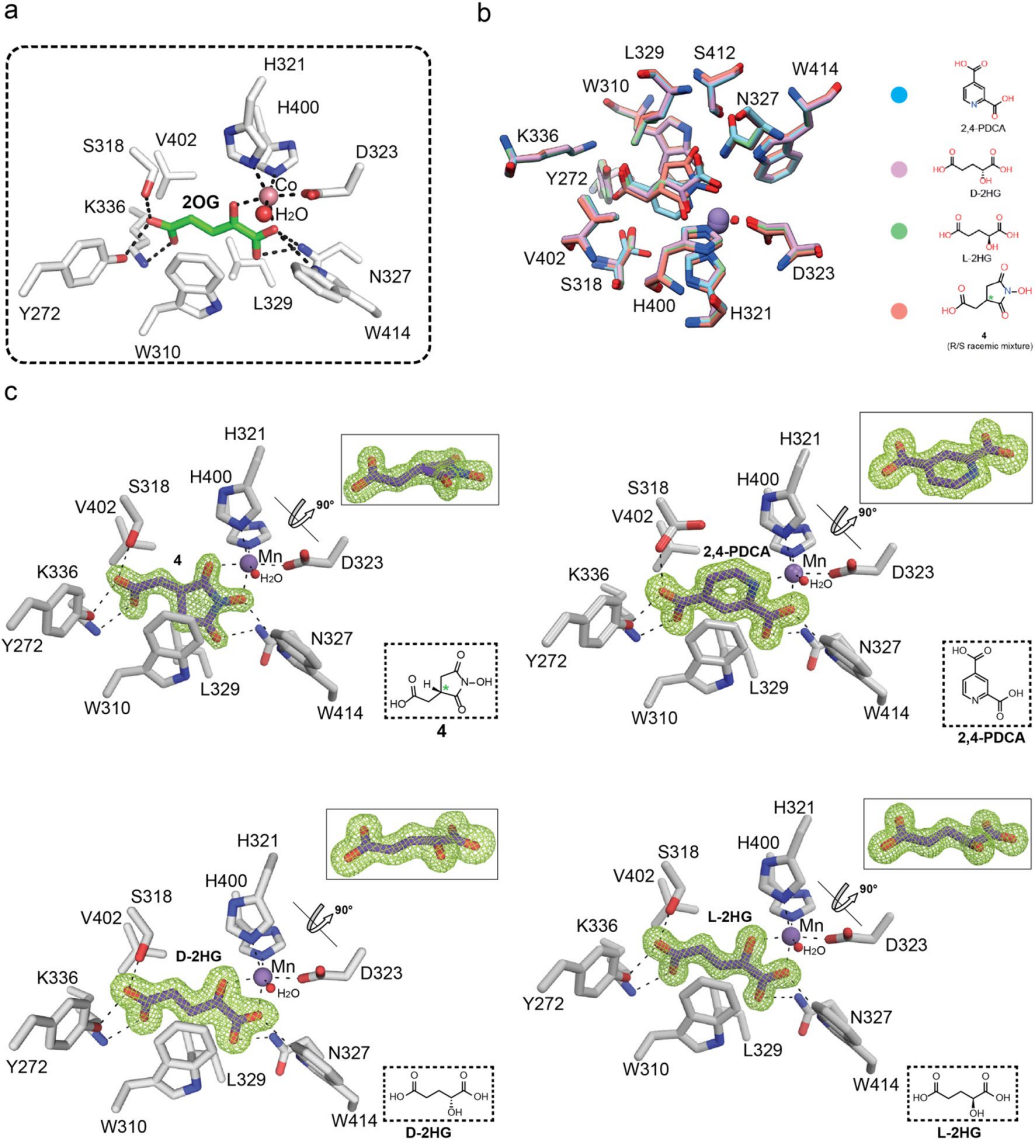
Views from crystal structures of JMJD5^∆1–181^ in complex with 2-oxoglutarate mimicking inhibitors. (**a**) Stick view of the active-site of JMJD5^∆1–181^ complexed with Co and 2OG (PDB: 4GJZ). (**b**) Overlay of crystal structure views of JMJD5^∆1–181^ in complex with the inhibitors D-2HG, L-2HG, **4**, and PDCA with individual complex structures shown in (**c**). (**c**) Views of crystal structures of JMJD5 in complex with **4**, PDCA, D-2HG, or L-2HG; the insets show 90° rotation following views of ligands along with their structures. Electron density OMIT maps for the inhibitors are contoured to 3σ. D-2HG = d-enantiomer of 2-hydroxyglutarate, L-2HG = l-enantiomer of 2-hydroxyglutarate. See Fig. S1 for structure of **4**. PDCA = pyridine-2,4-dicarboxylic acid. (*) indicates a chiral centre.

Previous crystallographic studies have revealed that some JmjC oxygenases, notably Factor Inhibiting HIF (FIH)^26^ and members of the JmjC KDM subfamily^40,41^ have relatively large and solvent accessible 2OG binding pockets. This feature can be exploited to obtain selectivity and increase the potency of 2OG mimetics^26^. To investigate the topology of the JMJD5 2OG binding pocket, we therefore carried out crystallographic analyses. We obtained high-resolution structures of JMJD5 comprising residues 182–416 (JMJD5^∆1–181^) in complex with catalytically inactive manganese (substituting for iron) and 2,4-PDCA (1.53 Å resolution), d-2HG (1.65 Å), l-2HG (1.36 Å) and the *N*-hydroxyimide **4** (1.49 Å) (Fig. 2, Supplementary Fig. S7). The structures were solved by molecular replacement using a reported JMJD5 structure (PDB: 4GJZ)^42^ as a search model and were in the *P*2_1_2_1_2_1_ space group, with a single JMJD5 molecule in the asymmetric unit.

Analyses of the difference density (*F*_o_-*F*_c_) maps around the active site in the four structures reveal that all the crystallised inhibitors coordinate the metal ion in a bidentate manner and bind in the JMJD5 2OG pocket (Fig. 2). As for 2OG itself, in each case, the C5 carboxylate (or equivalent carboxylate) is positioned to form an electrostatic interaction with K336 and to hydrogen bond with the alcohols of S318 and Y272 (Fig. 2). Indeed, the conformations of the C5 carboxylates are very well conserved in all the structures with the distances between inhibitor carboxylate and JMJD5 residues being (nearest non-hydrogen atoms): for 2OG: 2.6 Å/S318, 2.9 Å /Y272, 3.0 Å/K336; for **4:** 2.7 Å/S318, 2.7 Å/Y272, 2.8 Å/K336; for 2,4-PDCA: 2.8 Å/S318, 2.6 Å/Y272, 2.7 Å/K336; for d-2HG: 2.7 Å/S318, 2.6 Å/Y272, 2.7 Å/K336; and for l-2HG: 2.6 Å/S318, 2.7 Å/Y272, 2.7 Å/K336 (Supplementary Fig. S8).

As anticipated, in all the structures the catalytically inactive active site manganese (substituting for iron) is coordinated by the side chains of H321, D323, and H400 (Fig. 2). As is the case with the 2OG cosubtrate, a water molecule is ligated approximately *trans* to H400 in all the obtained inhibitor structures. Octahedral metal ion coordination is completed by bidentate coordination of the inhibitors with the C1 carboxylates of 2,4-PDCA, l-2HG, and d-2HG ligating *trans* to H321, and the alcohols of l-2HG, d-2HG, and pyridinium nitrogen of 2,4-PDCA ligating *trans* to D323 (which ligates in a monodentate manner) (Fig. 2).

The metal coordination mode of the cyclic inhibitor *N*-hydroxyimide **4** is of particular interest. Although structures of 2OG oxygenases in complex with hydroxamic acids have been reported, including for FIH^43^, there are no reported structures of them with cyclic hydroxamic acids or related compounds such as **4**. This is of particular interest in part because the natural product alahopcin^44,45^, which is a procollagen prolyl hydroxylase inhibitor is a cyclic hydroxamic acid and because approximately functionalised cyclic hydroxamic acids might be more selective inhibitors than acyclic ones such as Vorinostat/ SAHA, which also inhibits some JmjC KDMs^41^. Our structure of **4** with JMJD5 implies stereoselective binding of a single enantiomer (the 3*S*-enantiomer) despite use of a racemic mixture for crystallisation. The structure of JMJD5 complexed with **4** reveals that its ring is snugly bound in the 2OG pocket making hydrophobic interactions with the side chains of W310, L329, which normally interact with the 2OG methylenes (Fig. 2, Supplementary Fig. S8). The metal ion is coordinated by the *N*-hydroxy group oxygen (*trans* to H321) and one of the imide carboxyl oxygens (*trans* to D323).

Comparison of the binding modes of 2,4-PDCA and **4** at the JMJD5 active site reveals that the binding modes of the rings of the two inhibitors overlap (Fig. 2b), implying that modification of them to optimised potency should be possible and suggesting that binding of bicyclic inhibitors such as IOX1/ ML-324 might reflect an approximate composite of the binding modes of 2,4-PDCA and **4**.

The very similar binding modes of l-2HG and d-2HG (Fig. 2c) are of interest because l-2HG is a substantially more potent JMJD5 inhibitor (Table 1), showing that small differences in binding at the active site can make large differences in JMJD5 inhibition potency. The C2-methylenes of l-2HG are closer to the indole ring of W310 than are the C2-methylenes of d-2HG. Further, the *sp*3-hybridization at C2 of 2HG apparently forces the C3-C4 methylenes of l-2HG to adopt similar angular orientations as the C3-C4 methylenes of **4**, possibly reflecting the differences in potencies of l-2HG and d-2HG. Previous reports have shown that inhibition of human 2OG oxygenases by both l- and d-2HG is possible^35,46^; the concentration of d-2HG is elevated as a consequence of gain of function mutations to isocitrate dehydrogenases 1/2 (IDH 1/2)^47,48^. Mutations to the dehydrogenases converting l-2HG or d-2HG to 2OG can also result in elevations of l- or d-2HG levels^49^. Future work to systematically investigate the stereoselectivity of 2OG oxygenase inhibition by the 2HG enantiomers is therefore of interest.

The JMJD5 inhibition manifested by the clinically approved PHD inhibitor GSK1278863 (Daprodustat)^8,36^ is particularly interesting given its relatively large size. However, it is likely that the glycine side chain of GSK1278863 binds in the 2OG pocket with metal chelation by two of the carbonyl oxygens of its tricarbonyl unit^8^. The binding modes of the two cyclohexyl rings of GSK1278863 to JMJD5 is uncertain, but the fact that they can be accommodated close to the active site suggests that there is considerable scope for optimisation of GSK1278863 type tricarbonyl compounds for potent inhibition of JMJD5, as has been done for the 2OG oxygenase OGFOD1^50^.

The available JMJD5 crystal structures do not provide any evidence for induced fit binding of the inhibitors, as has been observed with some other 2OG oxygenases^38,51^; compared to the 2OG structure (PDB: 6F4N), the C_α_ RMSD is 0.229 for **4**, 0.240 for 2,4-PDCA, 0.273 for d-2HG, and 0.254 for l-2HG (Figure S8). However, preliminary modelling studies indicate that the binding of at least, the larger inhibitor molecules, including GSK1278863, ML-324, Vadadustat, 2.4-BPDCA and GSK-J1 (amongst others), will involve conformational changes of JMJD5 residues, in particular Q275 (located on the non-DSBH β-strand β5), W310 (βI of the DSBH), D323 (βII) W414 (βVIII) and enable different metal coordination modes (Supplementary Fig. S9).

### Comparison of JMJD5 structures with other JmjC-oxygneases

**Figure 3:**
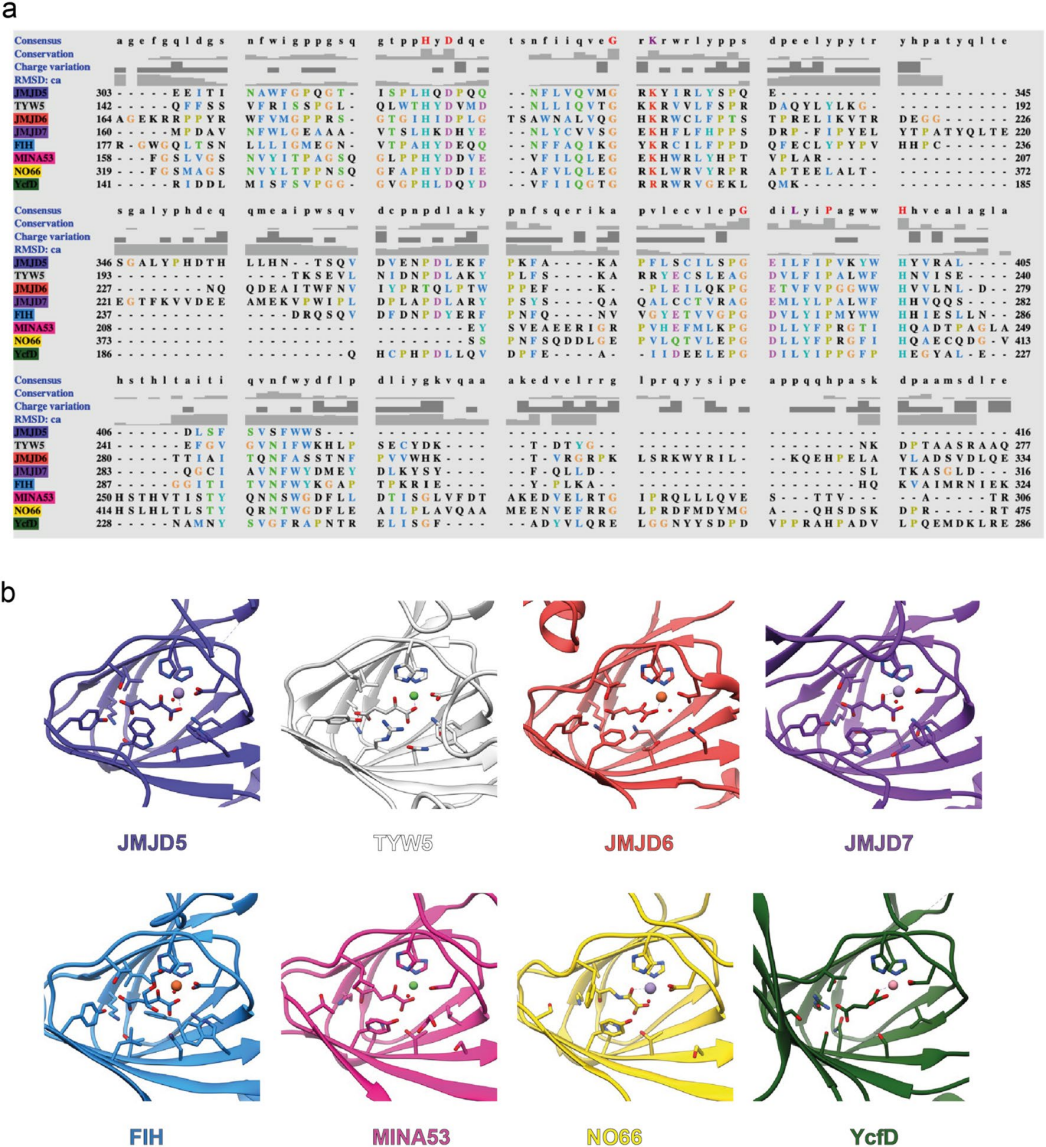
Ligand binding residues of JmjC hydroxylases. (**a**) Sequence-based alignment of JmjC domains of JMJD5 (UniProt-Q8N371), TYW5 (UniProt-A2RUC4), JMJD6 (UniProt-Q6NYC1), JMJD7 (UniProt-P0C870), FIH (UniProt-Q9NWT6), MINA53 (UniProt-Q8IUF8), NO66 (UniProt-Q9H6W3), and YcfD (UniProt-P27431) made using Clustal Omega in UCSF Chimera^76^. (**b**) Cartoon representation of the JmjC domain of 2OG hydroxylases with the ligand-binding residues in the site shown as sticks; JMJD5.Mn.2OG (PDB: 6F4N), TYW5.Ni.2OG (PDB: 3Al6), JMJD6.Fe.2OG (PDB: 6GDY), JMJD7.Mn.2OG (PDB: 5NFO), FIH.Fe.2OG (PDB: 4Z2W), MINA53.Ni.2OG (PDB: 4BU2), NO66.Mn.NOG (PDB: 4CCK), and YcfD.Co.2OG (PDB: 4LIT)**.** JMJD5^28^, MINA53^77^, NO66^77^ are human ribosomal oxygenases, YcfD is a bacterial ribosomal oxygenase^77^, TYW5 is a human tRNA wybutosine hydroxylase^78^, JMJD6 is a human lysine hydroxylase and is potentially a histone arginine demethylase^79–81^, JMJD7 is a reported human lysine hydroxylase and histone lysine/arginine demethylase^82,83^ and FIH is a human HIF asparaginyl hydroxylase^26,52,58,84^.

**Figure 4:**
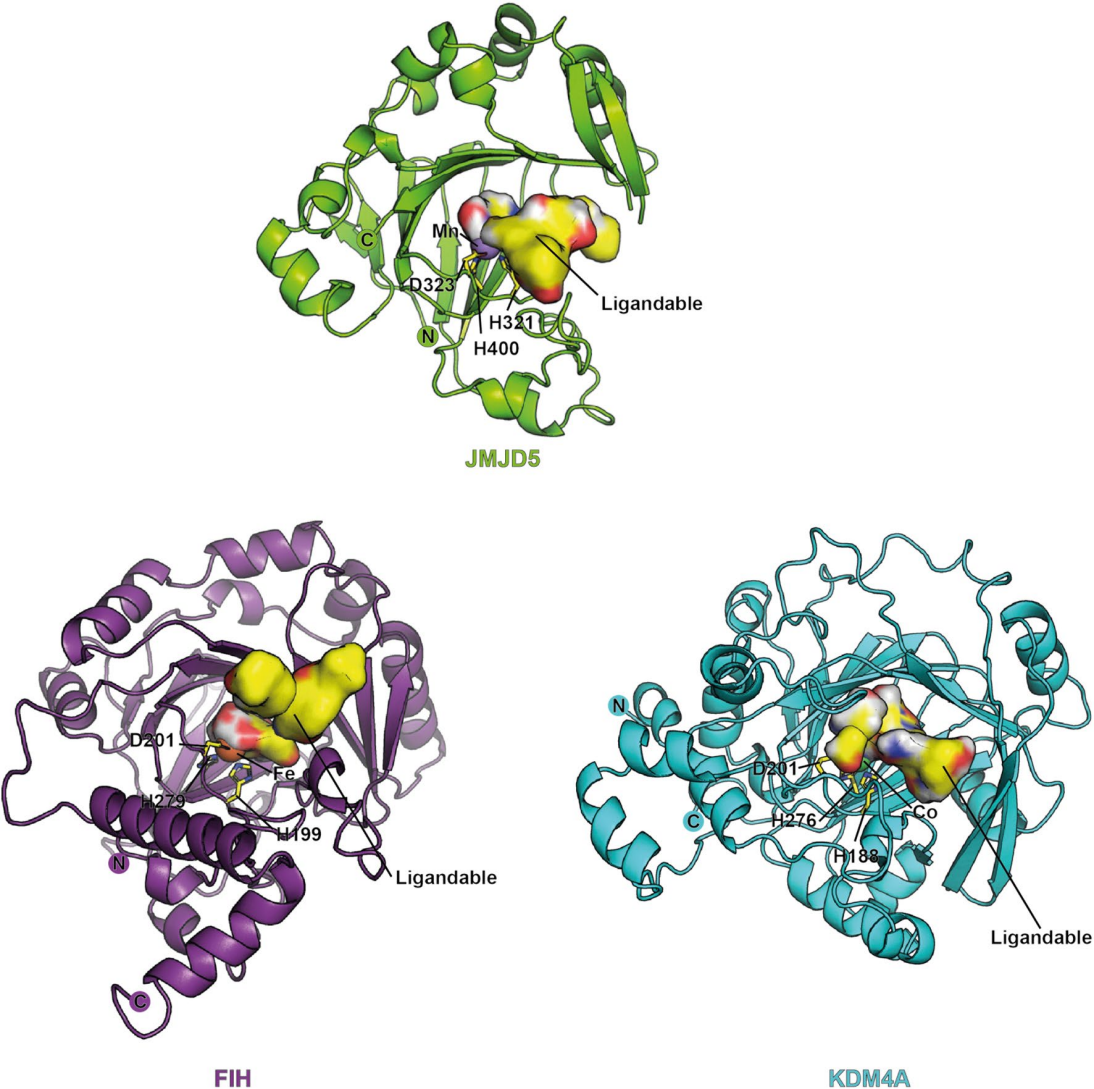
Comparisons of the 2-oxoglutarate binding pocket of JMJD5 with those of  FIH and KDM4A.

We compared the JMJD5 2OG/ NOG binding pocket with those of other JmjC oxygenases (Fig. 3, Supplementary Fig. S10 and Supplementary Fig. S11). A comparison of sequences of JmjC domains for which crystal structures are available reveals conservation of several key residues amongst human and bacterial JmjC hydroxylases (Fig. 3a). Analysis of the 2OG/NOG binding sites shows that many JmjC hydroxylases, including JMJD5, employ a similar set of residues for 2OG binding, though there are differences in detail and in their biological roles (Fig. 3b). Comparison of the JMJD5 ligand-binding residues with those of FIH^52^ (a representative and structurally well-studied JmjC hydroxylase) and KDM4A^53^ (a representative JmjC demethylase for which crystal structures are available) reveals that in each case, the 2OG C5 carboxylate (or inhibitor equivalent carboxylate) is bound by electrostatic interactions with a lysine (K214_FIH_, K206_KDM4A_ and K336_JMJD5_) and by hydrogen bonds with two residues with alcohol side chains (Y272_JMJD5_, S318_JMJD5_, add Y145_FIH_, T196_FIH_ and Y132_KDM4A_) (Supplementary Fig. S10). However, there is considerable variation in the other residues involved in the JmjC oxygenase 2OG binding pockets–in the case of JMJD5 these differences create a relatively tight 2OG binding pocket–the side chains of S318_JMJD5_, W310_JMJD5_, N327_JMJD5_, L329_JMJD5_, and V402_JMJD5_ are all positioned close to 2OG/the inhibitors (Fig. 4, Supplementary Fig. S10).

There are variations in the precise 2OG / 2OG competitor metal coordination and binding modes (Supplementary Fig. S10), depending on the oxygenase and 2OG competing ligands used in crystallographic studies. In each case, however, hydrophobic residues (as described above for JMJD5) are apparently involved in interaction with the 2OG methylenes, though there are considerable variations in the identity and conformations of these residues and, at least for some 2OG oxygenases including FIH, the conformations of some of these residues are different depending on the ligand bound^8,51^. Most strikingly, however, the 2OG binding site of JMJD5 is relatively compact compared to those of FIH and KDM4A (and many other 2OG oxygenases), rationalising the preference of JMJD5 for inhibition by 2OG competing compounds with relatively small groups binding in this pocket (Fig. 4, Supplementary Fig. S11).

By contrast with JMJD5, the ligand-binding groove of KDM4A is apparently substantially wider and deeper (Fig. 4, Supplementary Fig. S11)^53^. This may, at least in part, explain why inhibitors like 2,4-PDCA, 2,4-BPDCA and their structural analogues bind to KDM4A and to other JmjC KDMs more tightly than to JMJD5^54^. The available structural analyses reveal that binding of 2,4-PDCA involves more residues in the case of KDM4A (Y132_KDM4A_, Y177_KDM4A_, F185_KDM4A_, N198_KDM4A_, K206_KDM4A_, W208_KDM4A_, and K241_KDM4A_) than that for the JmjC-hydroxylases, FIH (Y145_FIH_, T196_FIH_, and K214_FIH_) and JMJD5 (Fig. S10). The relative lack of interactions made by 2,4-PDCA in the FIH and JMJD5 active sites is consistent with its apparently stronger inhibition of KDM4A (0.7 µM)^54^ versus JMJD5 (12 µM) and FIH (30 µM)^55^.

Neither daminozide (a plant growth regulator that inhibits the JmjC KDM 2/7 oxygenases^56^), nor its structural derivative **7** appear to inhibit JMJD5, even though their structures resemble that of NOG. Crystal structures of daminozide and substrates with KDM4A/PHF8 reveal a hydrophobic ligand-binding region at their active-site, which enables binding of the N-methyl amino groups of daminozide and substrates^53,56^. By contrast, the ligand-binding pocket of JMJD5 is relatively less hydrophobic in the analogous region (Fig. 4, Supplementary Fig. S11), potentially explaining why daminozide is a selective inhibitor of the KDM 2/7 JmjC subfamilies over JMJD5.

#### Discussion

JMJD5 is a particularly interesting JmjC hydroxylase because of its links to physiology, in particular circadian rhythm regulation in both plants and animals^31^ as well as in embryonic development^30^. Since genetic deletion of the JMJD5 gene shortens the circadian cycle, small-molecule JMJD5 inhibitors might be expected to behave similarly. The precise roles of JMJD5 in cells are unclear, both in terms of the reactions that it catalyses, the substrates it acts on, and the biopolymers with which it interacts. Research involving selective small-molecule modulation of JMJD5 activity will complement genetic approaches to defining its functions. Although there is clear scope for assay optimisation, our studies on the inhibition of JMJD5 will guide future structure-based work to develop potent and selective JMJD5 inhibitors. Work on Drosophila melanogaster, however, shows potential for involvement of JmjC oxygenases other than JMJD5 in circadian rhythm^57^, highlighting the need for selective inhibitors. In this regard, the cyclic tricarbonyl unit, as in the clinically used PHD inhibitor Daprodustat^36^, and the N-hydroxyimide scaffolds identified here are of particular interest for the development of selective JMJD5 inhibitors. The results should also help in development of selective inhibitors of other human oxygenases – development of PHD inhibitors is being slowed by observation of toxicity, potentially due to off target inhibition of other human 2OG oxygenases, such as JMJD5.

Hydroxamic acids are clinically used for the inhibition of human histone deacetylases and are being developed as inhibitors of other metallo-enzymes, though at least some of the histone deacetylase inhibitors also inhibit some JmjC KDMs^39^. Alahopcin is a naturally occurring cyclic hydroxamic acid that is an inhibitor of the procollagen prolyl hydroxylases (PHDs); simplified analogues of alahopcin have been shown to inhibit the hypoxia inducible factor prolyl hydroxylases^44,45^. However, to our knowledge cyclic hydroxamic acids and related compounds such as the *N*-hydroxyimide **4**, have not been developed as selective inhibitors of human metallo-enzyme. The structural information provided here should help enable the design of more potent and selective cyclic hydroxamate type inhibitors of metallo-enzymes.

The combined structural studies also demonstrate that the 2OG binding pocket of JMJD5 is unusual within JmjC 2OG oxygenase subfamily members in being relatively compact (Fig. 4, Supplementary Fig. S11). This observation is of interest both with respect to the development of JMJD5 inhibitors and inhibitors of other human 2OG oxygenases with less compact 2OG binding sites (including many, but not all, JmjC KDMs and hydroxylases)^6,8,19–26,37,38,41,43,44,51–53,55,56,58–62^ Whether or not the apparently unusually tight 2OG binding pocket of JMJD5 relates to its biological function is unclear, but our results suggest ways to test this by mutation of JMJD5 residues, including S318_JMJD5_, W310_JMJD5_, N327_JMJD5_, L329_JMJD5_, and V402_JMJD5._ In the case of the HIF prolyl hydroxylases (PHDs), we have proposed that tight binding of Fe and 2OG may help enable the PHDs to focus on hypoxia sensing/ variations in oxygen availability^59,62^. At least in the case of PHD2, these properties include the ability to form unusually stable complexes with 2OG and iron, as manifest by various biophysical analyses^63^.

We are presently working to optimise JMJD5 assays and to validate its natural substrates to investigate the possibility that its unusual 2OG binding pocket relates to its biological functions. Interestingly however, like JMJD5, PHD2 manifests only slow turnover of 2OG in the absence of a hydroxylation substrate^28^. The PHDs belong to a different structural subfamily compared to the JmjC 2OG oxygenases, where an arginine- rather than a lysine-residue is involved in binding the 2OG C5 carboxylate^60,64^. Given the unusual biochemical properties of PHD2 (at least of the PHDs) and that some JmjC KDMs are proposed as hypoxia sensors, the possibility that a member of the JmjC subfamily may have unusual 2OG binding properties is of interest.

## Materials and methods

### Expression and purification of recombinant JMJD5

DNA encoding for *N*-terminally His_6_-tagged full-length (aa 1–416) and truncated (aa 182–416) JMJD5 were inserted into the expression vector pNIC28-Bsa4 (GeneBank ID:EF198106) and were expressed in *Escherichia coli* Rosetta2(DE3)-pLysS cells. Cells were grown in the 2x-tryptone/yeast extract (2TY) media supplemented with 30 µg/mL kanamycin and 34 µg/mL chloramphenicol at 37 °C to an OD_600_ = 0.6. JMJD5 protein production was induced by adding 0.5 mM β-d-1-isopropyl thiogalactopyranoside (IPTG) at 18 °C overnight; the cells were harvested by centrifugation at 4000 × g for 8 min) and were stored at −80 °C. The cell pellets were resuspended in the lysis buffer (50 mM HEPES pH 7.5, 500 mM NaCl, 10 mM imidazole, 5% glycerol, 4 mM MgCl_2_, ethylenediaminetetraacetic acid (EDTA)-free protease inhibitor cocktail tablet (Roche), and DNaseI (bovine pancreas, grade II, Roche) and lysates were loaded on to a 5 mL HisTrap column (GE Healthcare) that has been equilibrated with the binding buffer (50 mM HEPES pH 7.5, 500 mM NaCl, 10 mM imidazole, and 5% (v/v) glycerol using an AKTA purifier (GE Healthcare). The column was washed with 50 mM HEPES pH 7.5, 500 mM NaCl, 60 mM imidazole, and 5% (v/v) glycerol. JMJD5 was eluted with 50 mM HEPES pH 7.5, 500 mM NaCl, 500 mM imidazole, and 5% (v/v/) glycerol using a linear gradient (0–100%). Based on the UV-trace (at 280 nm) and sodium dodecyl sulphate–polyacrylamide gel electrophoresis (SDS-PAGE) analysis, fractions containing JMJD5 were pooled and concentrated to 2 mL. The so obtained protein was further purified using a Superdex 200 size-exclusion column (GE Healthcare) using the size-exclusion buffer (50 mM HEPES pH 7.5, 200 mM NaCl, and 5% (v/v) glycerol). The desired fractions (based on UV-traces and SDS-PAGE analysis) were concentrated to 25–30 mg/mL of JMJD5, then flash froze using liquid nitrogen and stored at −80 °C for future use.

### Inhibition Assay by MALDI-TOF MS

JMJD5 inhibition assays were carried out by monitoring the reaction between 10 µM full-length JMJD5 (aa 1–416) and 100 µM RpS6_129-144_ (VPRRLGPK**R**ASRIRKL) in the presence of an inhibitor (varied concentrations) at 37 °C in 50 mM HEPES-Na pH 7.5 buffer supplemented with 100 µM (NH_4_)_2_Fe(SO_4_)_2_.6H_2_O (SIGMA-Aldrich), 400 µM l-sodium ascorbate (SIGMA-Aldrich), and 500 µM 2OG (Fluka). For end-point inhibition assays, inhibitors were used at 0.1, 1, and 10 mM and reactions were quenched after 30 min. Hydroxylation of RpS6 peptide was monitored by MALDI-TOF MS and intensity of hydroxylated peak was quantified to determine the extent of inhibition caused by inhibitor. For IC_50_ determinations, the same in vitro assay reaction was used; a dose–response curve for each inhibitor was constructed using 6 concentrations of inhibitor (0.05–10 mM) and Prism 9 (GraphPad) was used to calculate IC_50_ values.

### Crystallography

JMJD5^**Δ1–181**^ of ≥ 95% purity (confirmed by SDS-PAGE analysis) and at a concentration of ≥ 25 mg/mL was used for crystallisation. JMJD5.Mn.inhibitor complexes were formed by incubating the enzyme with the other components (usually in fivefold excess) on ice for ~ 1 h. A Phoneix™ RE crystallization robot (Art Robbins Instruments) and a Minstrel-HT™ platform with CrystalTrek™ from Rigaku Inc. were used to set up crystallisation plates. Crystals were allowed to grow by sitting drop vapour diffusion method (drop size, 200–300 nL) in 96-well intelli-plates (Art Robbins) at 25 °C. JMJD5.Mn.inhibitor complexes were crystallised in 0.1 M Bis–tris pH 6.5, 15% (w/v) PEG3350, and 0.002 M MnCl_2_. Crystals were cryo-protected using a mixture of reservoir solution diluted with 25% (v/v) glycerol. Diffraction data were collected at the DLS (Diamond Light Source) synchrotron and processed by HKL2000^65^ or alternatively CCP4 package software^66^. The structures were solved by molecular replacement using PHASER^67^ and PDB ID: 4GJZ as the search model. All four JMJD5.inhibitor complex structures were solved in the *P*2_1_2_1_2_1_ space group and structural models were improved by iterative cycles of manual building in COOT^68^ and refinement in PHENIX^69,70^.

### Modelling and computational studies

JMJD5.inhibitor modelled complexes were generated using JMJD5 coordinates from PDB ID: 4GJZ, with ligands (GSK1278863, ML-324, Vadadustat, GSK-J1 and 2.4-BPDCA) docked using AutoDock Vina^71^. An initial model was prepared by using AutoDock Tools: non-protein ligands and all crystallographic waters were removed; all residues were in their default protonation states, polar H atoms were added, histidines were protonated at the delta position, Kollman-united charges were applied to the protein. AutoGrid4 was used to define the search space/ docking site on JMJD5 that included a box size of 24 Å × 16 Å × 16 Å, centred around the active site metal with x, y, z coordinates of 10.603, 11.622, 25.082. We tested the grid centre and the optimized box size by re-docking 2,4-PDCA and **4**, followed by comparing their lowest energy states with the respective crystal structures (PDB ID: 6I9L and 7UQ3). The docking tools were configured to employ a rigid protein, flexible ligands along with the above grid box properties to generate maximum 9 poses for each ligand using AutoDock Vina v1.1.2. Docking poses in which ligands did not maintain octahedral coordination with the metal centre were excluded from further analyses. Selected models were validated by comparing with the orientations of same/ similar ligands as observed in the crystal structures of structurally related 2OG oxygenase complexes; the following PDB IDs were used for comparison / validation purposes: PDB: 5OX5 (PHD2.CCT6 complex)^8^ for GSK1278863; PDB: 4BIS (KDM4A.8HQ)^38^ for ML-324; PDB: 5OPC (FIH.Vadadustat)^8^ for Vadadustat, PDB: 4ASK (JMJD3.GSK-J1)^72^ for GSK-J1 and PDB: 6I9L (JMJD5.2,4-PDCA) for 2,4-BPDCA. All modelled complexes including appropriate crystallographic waters were finally conjugate energy minimized using phenix.geometry_minimization^73^ without applying external energy terms. Parameter and topology files for the ligands for energy minimization were generated using PRODRG^74^. FTMap was used to calculate ligand-binding sites^75^.

## Supplementary Information

Supplementary Information.

## Data Availability

Coordinates and structure factors for the JMJD5.inhibitor complex structures are deposited in the RCSB Protein Data bank as: JMJD5.Mn.2,4-PDCA (PDB ID: 6I9L), JMJD5.Mn.D-2HG, PDB ID: 6I9M, JMJD5.Mn.L-2HG, PDB ID: 6I9N and JMJD5.Mn.**4**, PDB ID: 7UQ3.
