# Supplementary material for: Structural analysis of the 2-oxoglutarate binding site of the circadian rhythm linked oxygenase JMJD5
Source: Sci Rep. 2022 Nov 30;12:20680. doi: 10.1038/s41598-022-24154-0 (PMC9712658; doi:10.1038/s41598-022-24154-0)
Supplement: Supplementary file 1 — Supplementary Information. [file 41598_2022_24154_MOESM1_ESM.pdf]

Supplementary Information

Structural Analysis of the 2-Oxoglutarate Binding Site of the Circadian Rhythm Linked ~~O~~oxygenase JMJD5

Md. Saiful Islam, Marios Markoulides, Rasheduzzaman Chowdhury*, and Christopher J. Schofield*

Chemistry Research Laboratory, Department of Chemistry and the Ineos Oxford Institue for Antimicrobial Research, University of Oxford, 12 Mansfield Road, Oxford, OX1 3TA, United Kingdom.

* Emails for correspondence: chowdhury.rzaman@gmail.com or christopher.schofield@chem.ox.ac.uk

Table S1. Crystallographic data collection and refinement statistics of JMJD5.inhibitor crystal structures.

Figure S1. Chemical structures of compounds used in the inhibition studies of JMJD5.

Figure S2. Inhibitor screening against human JMJD5.

Figure S3. IC_{50} curves of JMJD5 inhibition by 2OG mimetics.

Figure S4. IC_{50} curves of JMJD5 inhibition by TCA cycle intermediates.

Figure S5. IC_{50} curves for JMJD5 inhibition by selected PHD inhibitors.

Figure S6. IC_{50} curves for JMJD5 inhibition by selected KDM inhibitors.

Figure S7. JMJD5.inhibitor complex crystal structure views.

Figure S8. Comparison of JMJD5.inhibitor complex structures.

Figure S9. Inhibitor induced conformational changes in the JMJD5 active site.

Figure S10. Comparison of inhibitor-complexed active-sites of JMJD5, FIH and KDM4A.

Figure S11. Comparisons of the 2-oxoglutarate binding pockets of JMJD5, FIH and KDM4A.

Figure S12. Overview of phylogenetic relationships and domain architectures of human 2OG oxygenases.

Scheme 1. Syntheses of **4** and **5**.

Figure S13. NMR spectra of di-*tert*-butyl 2,2'-(oxalylbis(azanediyl))diacetate.

Figure S14. NMR spectra of oxamide-*N*,*N*-diacetic acid.

Table S1. Crystallographic data collection and refinement statistics of JMJD5.inhibitor crystal structures.

	JMJD5.Mn(II). **4**	JMJD5.Mn(II). 2,4-PDCA	JMJD5.Mn(II). D-2HG	JMJD5.Mn(II). L-2HG
PDB Acquisition Code	7UQ3	6I9L	6I9M	6I9N
X-Ray Source	DLS I02	DLS I04-1	DLS I02	DLS I04-1
Wavelength (Å)	0.9800	0.9174	0.9800	0.9174
Resolution (Å)	78.23-1.49 (1.52-1.49)*	50.00-1.53 (1.58-1.53)*	41.75-1.65 (1.71-1.65)*	50.00-1.36 (1.41-1.36)*
Space Group	$P2_12_12_1$	$P2_12_12_1$	$P2_12_12_1$	$P2_12_12_1$
Unit Cell Dimensions				
a, b, c	49.4, 64.6, 78.2	49.5, 64.8, 77.9	49.5, 64.6, 77.4	49.6, 64.4, 77.5
α, β, λ	90, 90, 90	90, 90, 90	90, 90, 90	90, 90, 90
Molecules per ASU	1	1	1	1
Wilson B Factor (A^2)	14.0	12.5	18.2	14.5
Reflections	41815 (2058)*	38444 (3781)*	30597 (2984)*	53414 (5302)*
Completeness	100 (100)*	99.6 (99.7)*	99.8 (99.2)*	98.8 (99.4)*
Multiplicity	7.0 (7.0)*	6.2 (5.6)*	6.7 (6.2)*	9.6 (8.9)*
$I/\sigma I$	11.0 (2.7)*	17.6 (6.6)*	18.17 (3.18)*	24.1 (4.2)*
R_{sym}	0.093 (0.773)*	0.119 (0.55)*	0.108 (0.78)*	0.081 (0.529)*
CC half	0.996 (0.785)*	0.989 (0.898)*	0.995 (0.765)*	0.997 (0.934)*
R_{cryst}	0.134	0.146	0.151	0.149
R_{free}	0.135 (5.0)[¥]	0.164 (4.9)[¥]	0.181 (4.9)[¥]	0.158 (4.9)[¥]
Deviation from Idealized Geometry				
Bond Length (Å)	0.009	0.008	0.018	0.008
Bond angles (°)	1.460	0.949	1.400	1.000
Average B Factors				
All atoms	18.7	18	21.5	23.7
Protein	16.7	7.2	19.3	21.8
Ligand	16.4	15.8	12.9	15.3
Metal	10.2	8.2	11.6	12.6
Water	31.4	31.8	35.9	38.4
Ramachandran Plot				
Favoured (%)	97.8	99.2	98.3	98.4
Allowed (%)	2.2	0.8	1.7	1.6
Disallowed (%)	0.0	0.0	0.0	0.0

* Highest resolution bin.
[¥] Percentage of the total reflections used for R_{free} calculations.

Figure S1. Chemical structures of compounds used in the inhibition studies of JMJD5.

a. 2OG Mimetics, Pyridines, 8-Hydroxyquinolines, and Daminozide

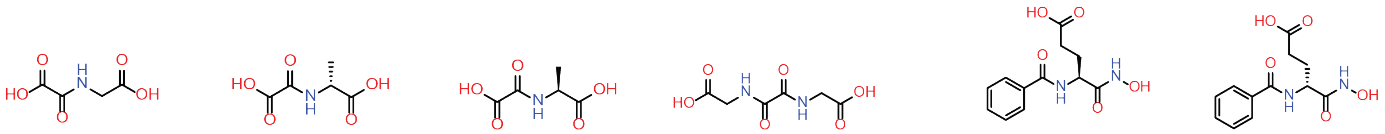

NOG | N-oxalyl-D-Alanine (NODA) | N-oxalyl-L-Alanine (NOLA) | **1** | **2** | **3**

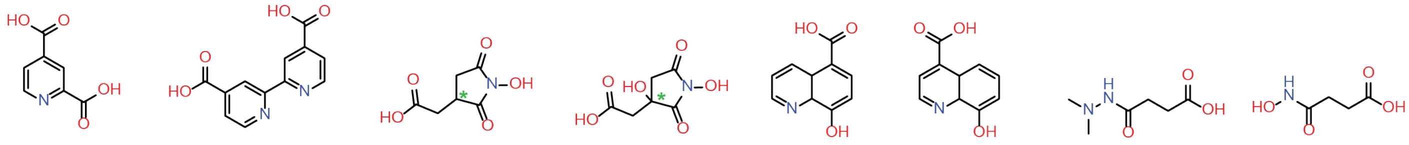

2,4-PDCA | 2,2'-BP-4,4'-DCA | **4** (R/S racemic mixture) | **5** (R/S racemic mixture) | IOX1 | **6** | Daminozide | **7**

b. TCA Cycle Intermediates

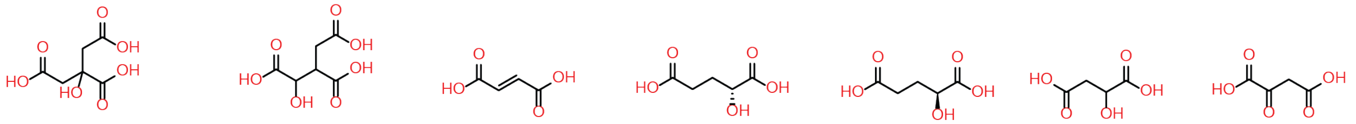

Citrate | Isocitrate | Fumarate | D-2HG | L-2HG | L-Malate | Oxaloacetate

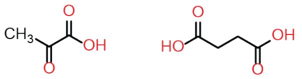

Pyruvate | Succinate

c. PHD Inhibitors

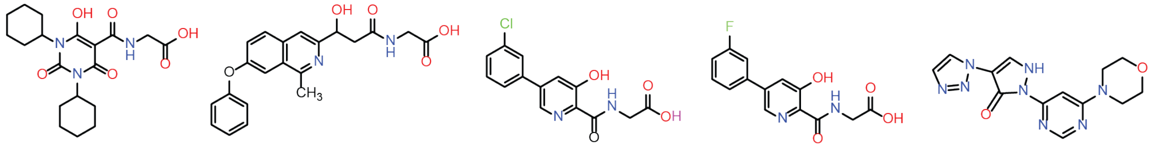

GSK1278863/Daprodustat | FG4592 | Vadadustat | AKB-6899 | Molidustat

d. KDM Inhibitors

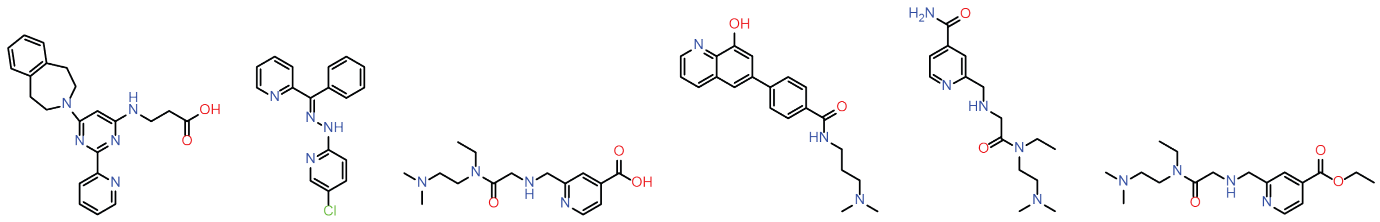

GSK-J1 | JIB-04 | KDM5-C49 | ML-324 | KDOAM25 | KDM5-C70

Figure S2. Inhibitor screening against human JMJD5.

a

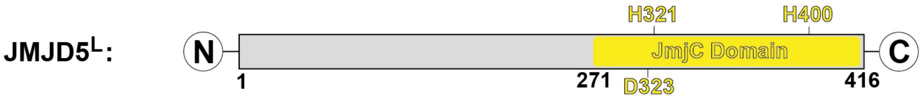

b

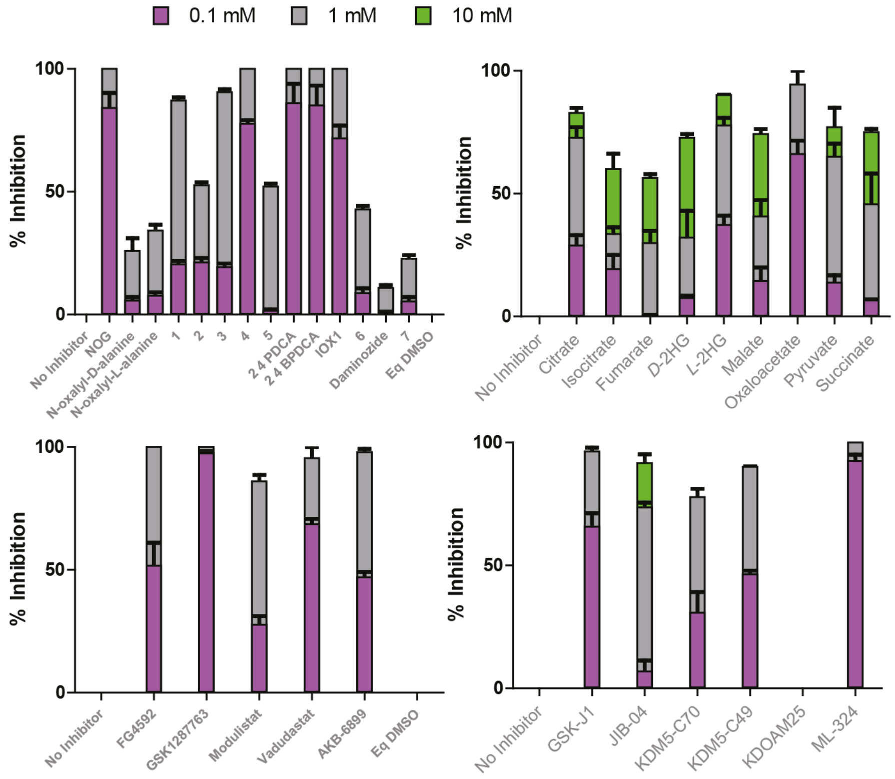

(a) Domain topology of full-length construct of JMJD5 (aa 1-416, JMJD5L) that was used in the inhibitor screening. (b) End-point inhibition of JMJD5 by 2OG mimetics, TCA cycle intermediates, selected PHD inhibitors, and selected KDM inhibitors. Concentrations of inhibitors used in this screening assay were 0.1, 1, and 10 mM. Values represent mean (n = 3) ± SD. Linked to tables 1 and S1, figures 2-4 and S3-S6.

Figure S3. IC_{50} curves of JMJD5 inhibition by 2OG mimetics.

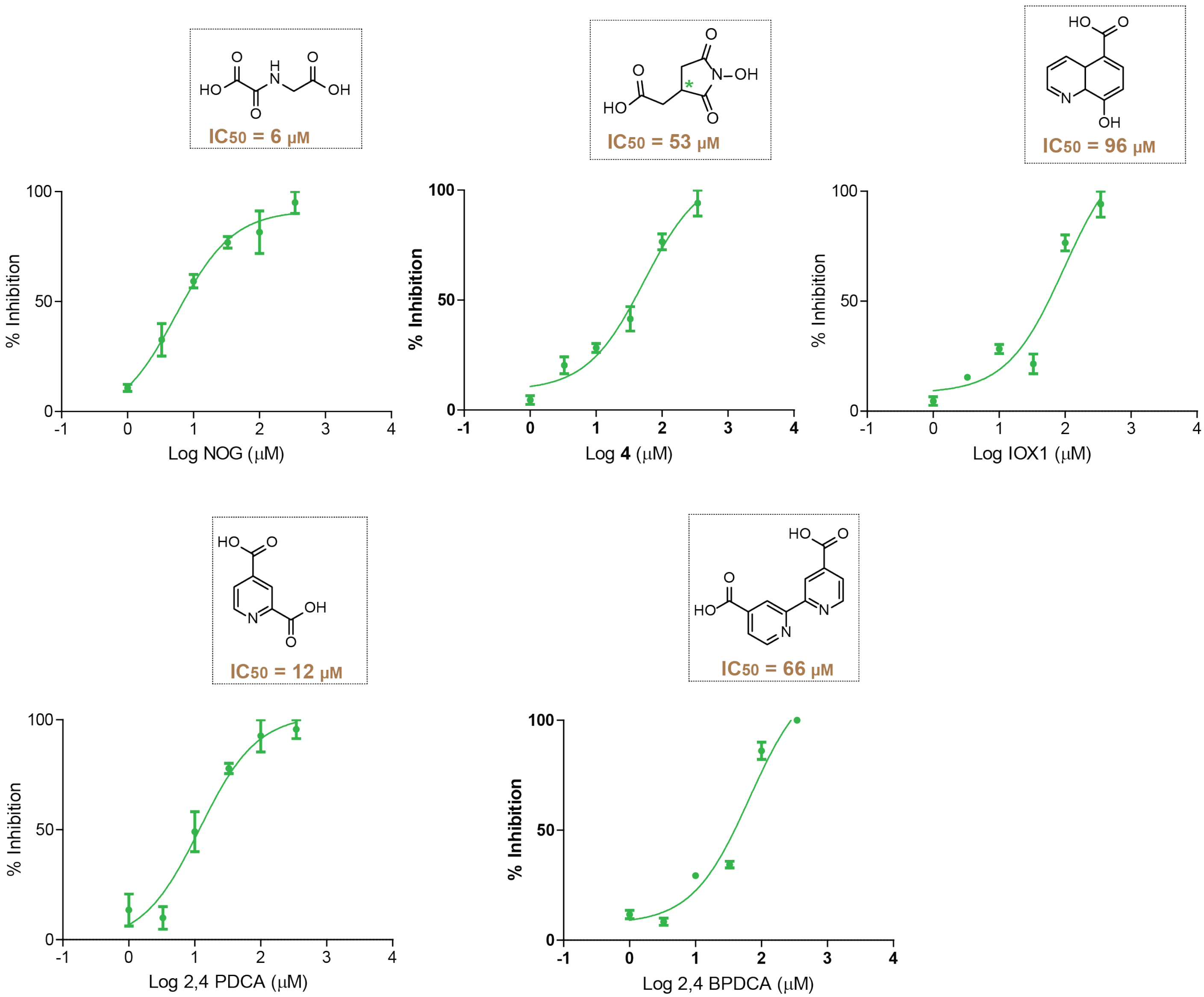

See legend to Table 2 for assay conditions. Note complete inhibition was not observed at the highest tested concentration for all compounds. Linked to tables 1 and S1, figures 2-4.

Figure S4. IC_{50} curves of JMJD5 inhibition by TCA cycle intermediates.

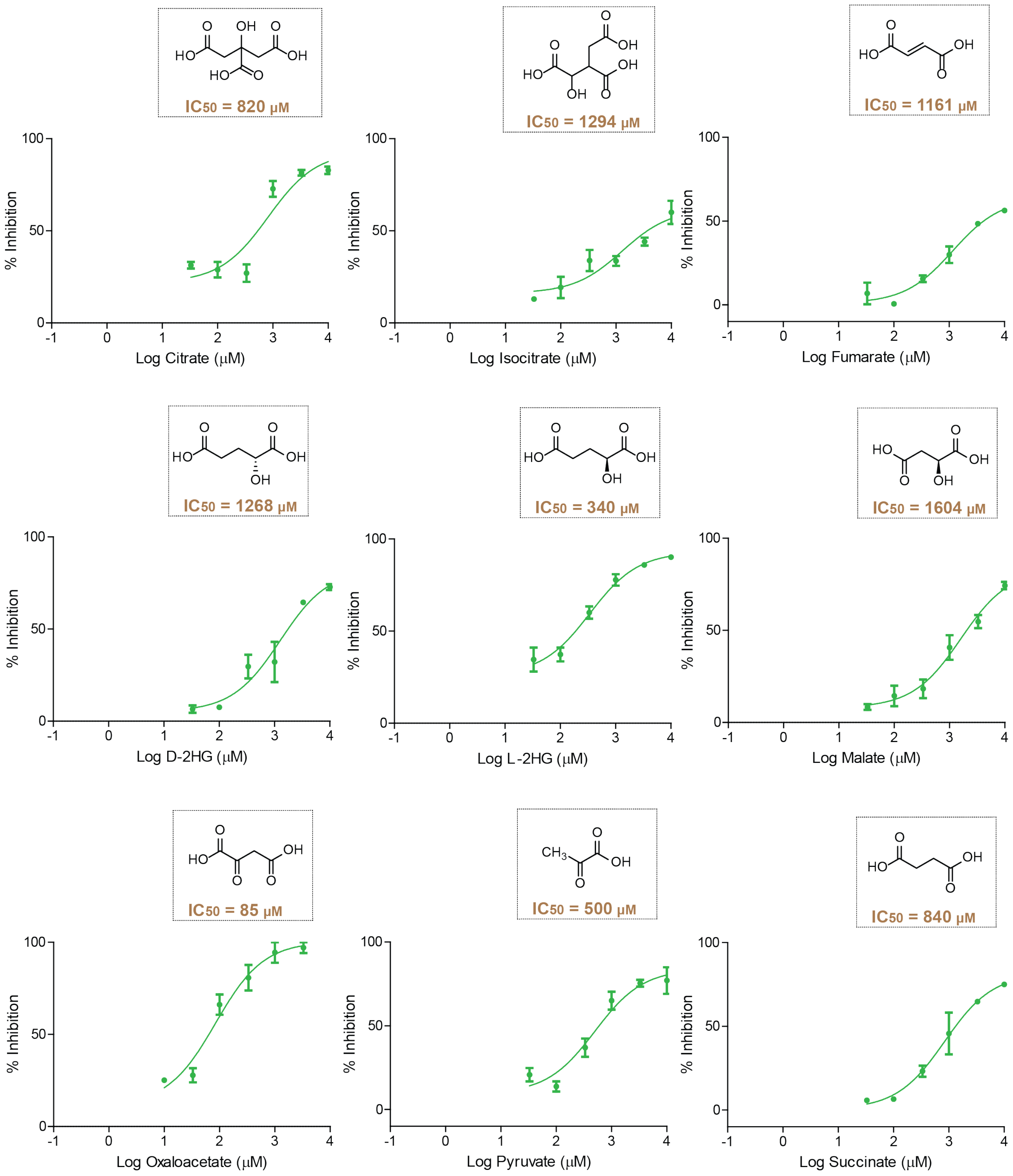

See legend to Table 2 for assay conditions. Note complete inhibition was not observed at the highest tested concentration for all compounds. Linked to tables 1 and S1, figures 2-4.

Figure S5. IC_{50} curves for JMJD5 inhibition by selected PHD inhibitors.

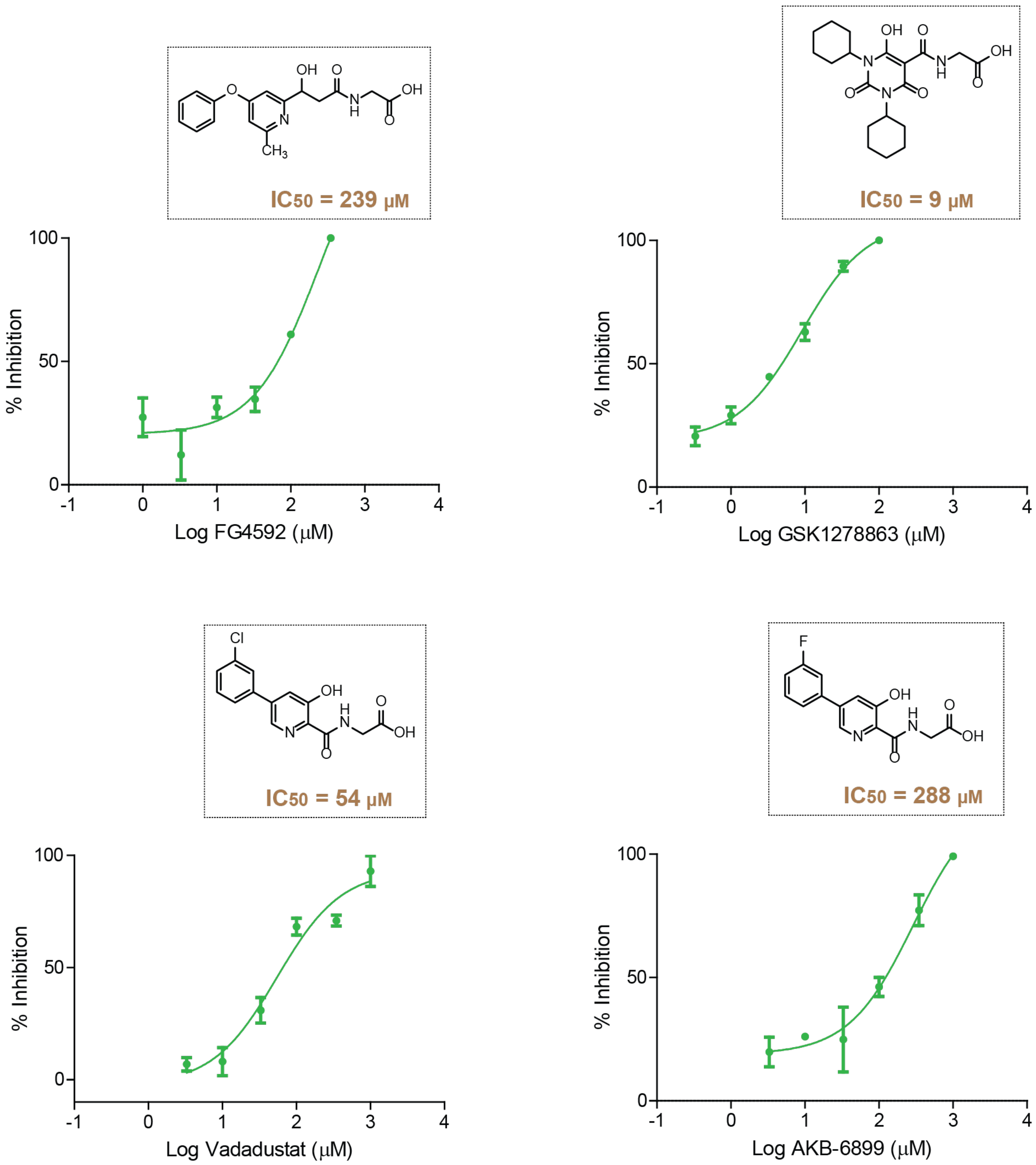

See legend to Table 1 for assay conditions. Note complete inhibition was not observed at the highest tested concentration for all compounds. Linked to tables 1 and S1, figures 2-4.

Figure S6. IC_{50} curves for JMJD5 inhibition by selected KDM inhibitors.

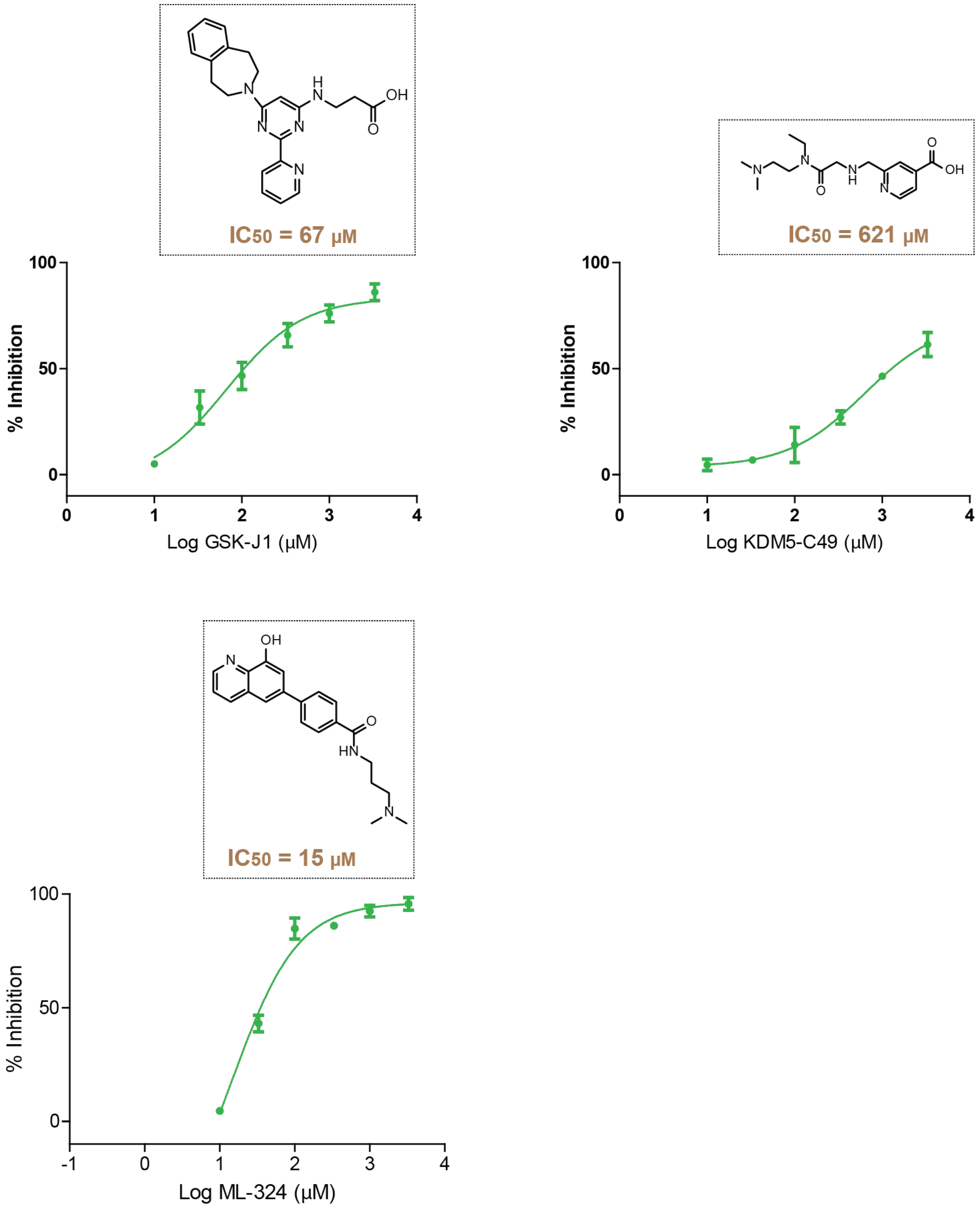

See legend to Table 1 for assay conditions. Note complete inhibition was not observed at the highest tested concentration for all compounds. Linked to tables 1 and S1, figures 2-4.

Figure S7. JMJD5.inhibitor complex crystal structure views.

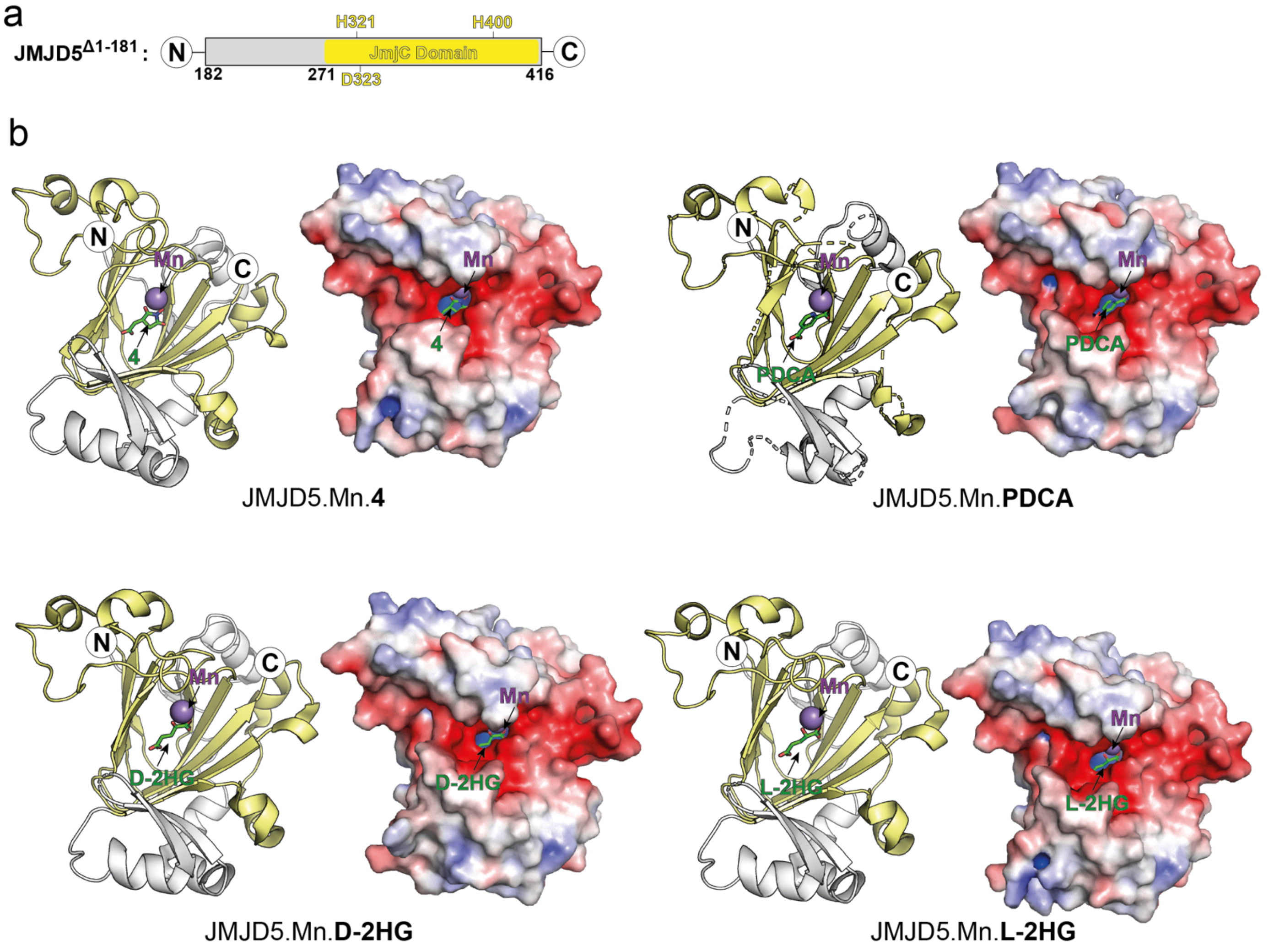

(a) Domain topology of a truncated version of JMJD5 with the catalytic JmjC domain (yellow) (JMJD5$^{\Delta 1\text{-}181}$). (b) Cartoon and surface (in PyMOL) presentations of crystal structures of JMJD5$^{\Delta 1\text{-}181}$ in complex with its identified inhibitors **4**, PDCA, D-2HG, and L-2HG. D-2HG = D-isoform of 2-hydroxyglutarate. L-2HG = L-enantiomer of 2-hydroxyglutarate. For the structure of **4** see Figure S1. PDCA = pyridine-2,4-dicarboxylic acid.

Figure S8. Comparison of JMJD5.inhibitor complex structures.

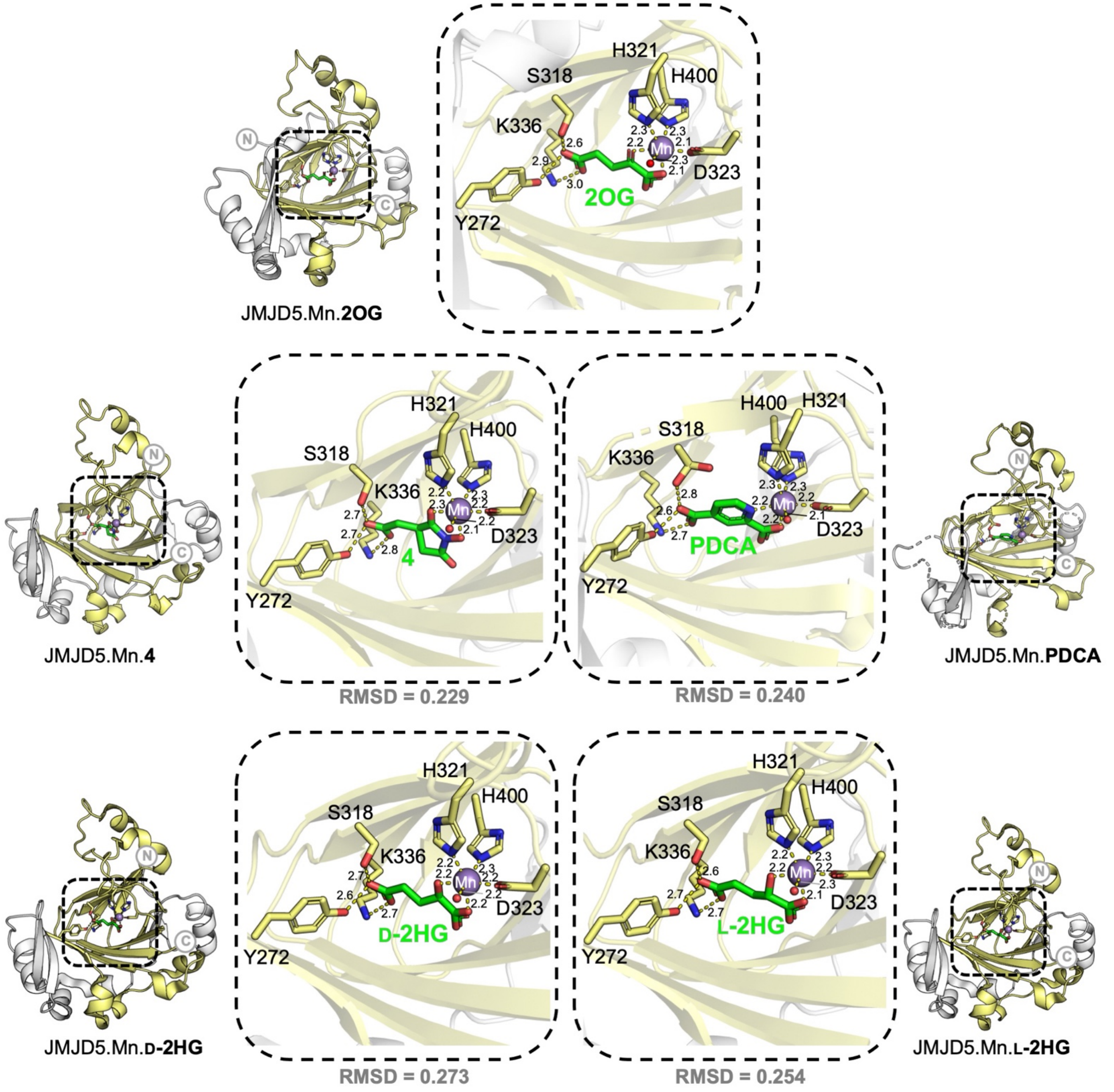

Cartoon presentation of crystal structures of JMJD5 in complex with 2OG (PDB: 6F4N), **4**, PDCA, D-2HG, and L-2HG, showing C_{α} RMSD (root-mean-square deviation) and distances of C5-carboxylate from metal. D-2HG = D-isoform of 2-hydroxyglutarate. L-2HG = L-enantiomer of 2-hydroxyglutarate. For the structure of **4** see Figure S1. PDCA = pyridine-2,4-dicarboxylic acid (see Figure S1).

Figure S9. Inhibitor induced conformational changes in the JMJD5 active site.

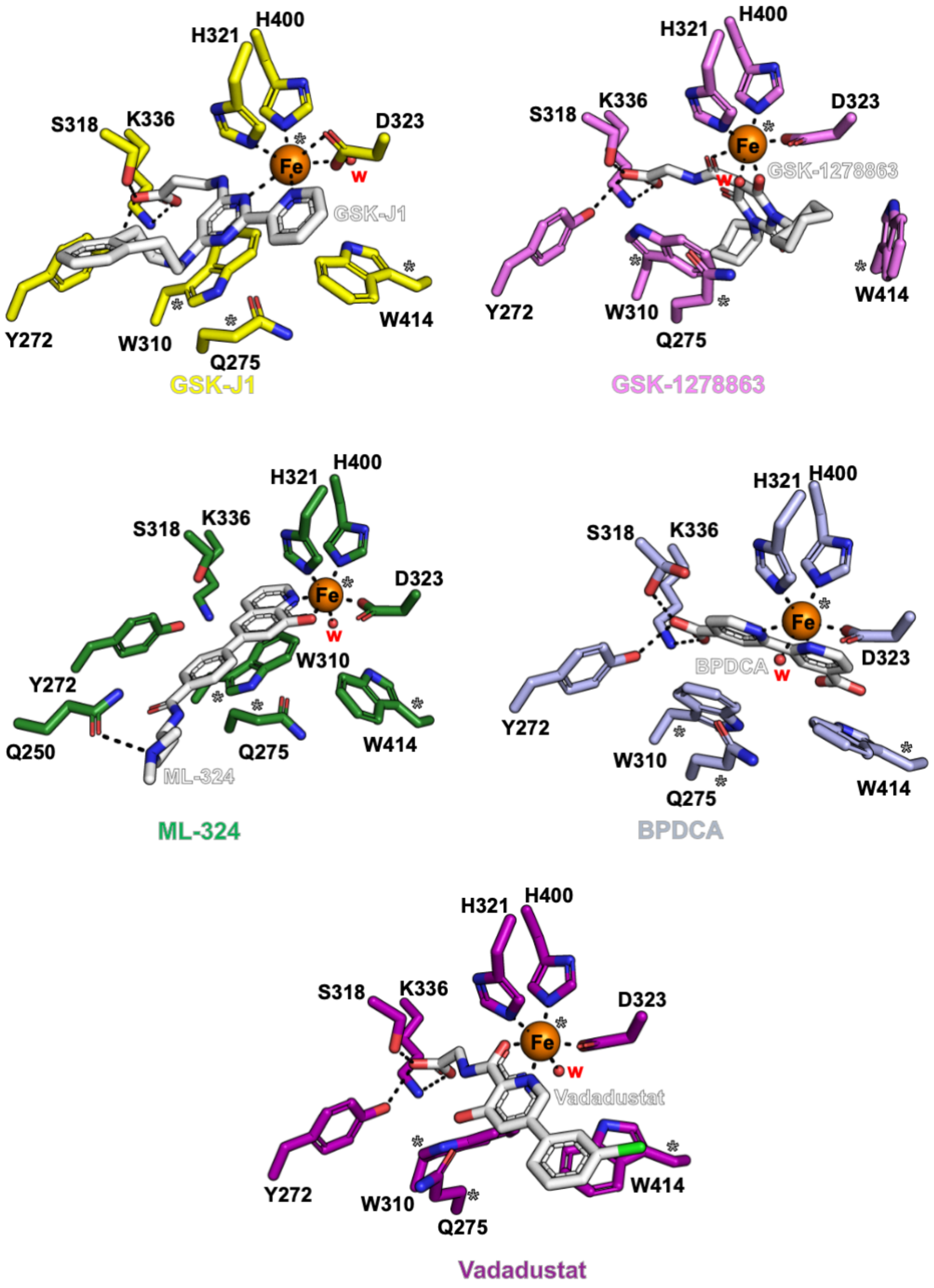

Stick presentation of JMJD5 catalytic domain with its identified inhibitors modelled (AutoDock Vina) at its active-site, showing inhibitors with larger chemical groups like GSK-J1, GSK-1278863, ML-324, BPDCA, and Vadadustat can induce conformational changes in the active-site region. BPDCA = 2,2-bipyridine-4,4- dicarboxylic acid.

Figure S10. Comparison of inhibitor-bound active-sites of JMJD5, FIH and KDM4A.

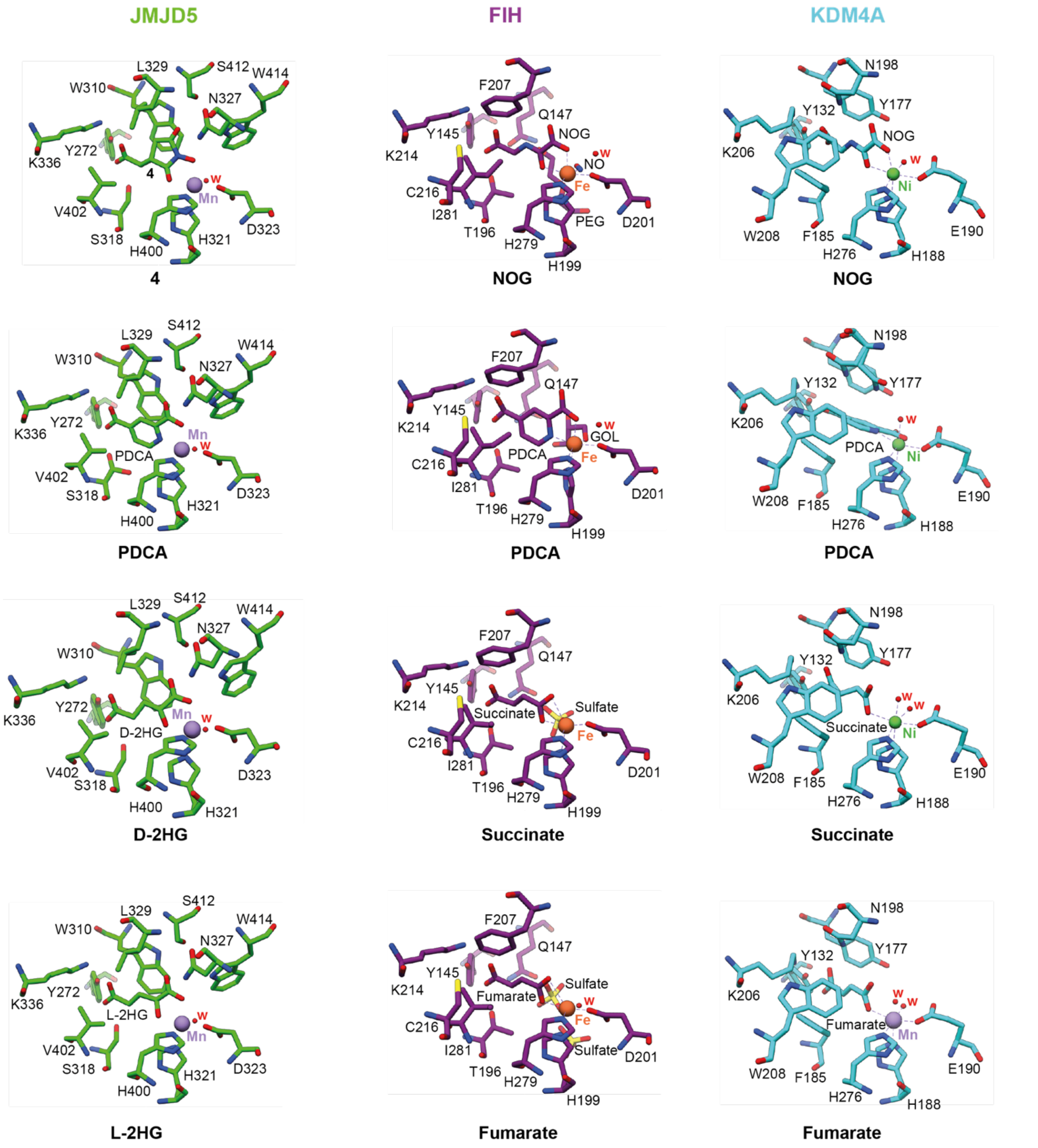

Views from crystal structures of JMJD5 (green), a JmjC hydroxylase FIH (purple) and a JmjC demethylase KDM4A (cyan) showing residues involved in 2OG / 2OG mimetic binding in the active site. PDBs: 4Z1V (FIH.Fe.NOG), 2W0X (FIH.Fe.PDCA), 2CGN (FIH.Fe.succinate), 2CGO (FIH.Fe.Fumarate), 2OQ7 (KDM4A.Ni.NOG), 2VD7 (KDM4A.Ni.PDCA), 5FYC (KDM4A.Ni.succinate), and 5FYH (KDM4A.Mn.Fumarate). D-2HG = D-enantiomer of 2-hydroxyglutarate, L-2HG = L-enantiomer of 2-hydroxyglutarate. See Figure 2 for the structure of **4**. PDCA = pyridine-2,4-dicarboxylic acid.

Figure S11. Comparison of the 2-oxoglutarate binding pockets of JMJD5, FIH and KDM4A.

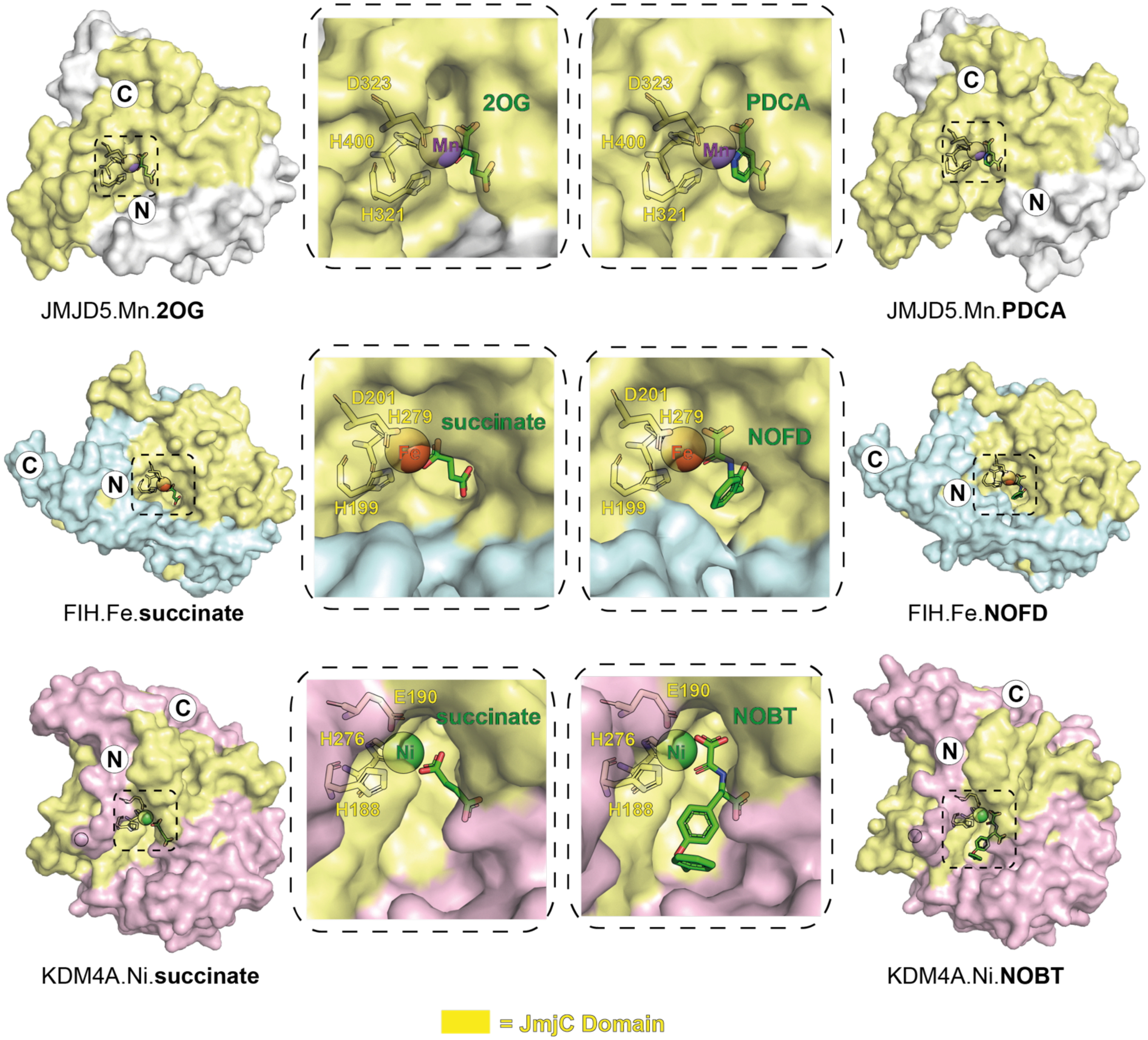

Surface presentations of inhibitor-bound crystal structures of JMJD5, FIH, and KDM4A; the insets show the 2OG/ligand-binding pocket, which in case of JMJD5 is the smallest and in KDM4A is the largest. The JmjC domain is in pale yellow and the rest of the structure is in white (JMJD5), pale cyan (FIH), and light pink (KDM4A). PDCA = pyridine-2,4-dicarboxylic acid, NOFD = *N*-oxalyl-D-phenylalanine, NOBT = *N*-oxalyl-D-(O-benzyl)tyrosine. PDB IDs: 6F4N (JMJD5.Mn.2OG, 6I9L (JMJD5.Mn.PDCA), 2CGN (FIH.Fe.succinate), 1YCI (FIH.Fe.NOFD), 5FYC (KDM4A.Ni.succinate), and 2WWJ (KDM4A.Ni.NOBT).

Figure S12. Overview of phylogenetic relationships and domain architectures of human 2OG oxygenases.

a

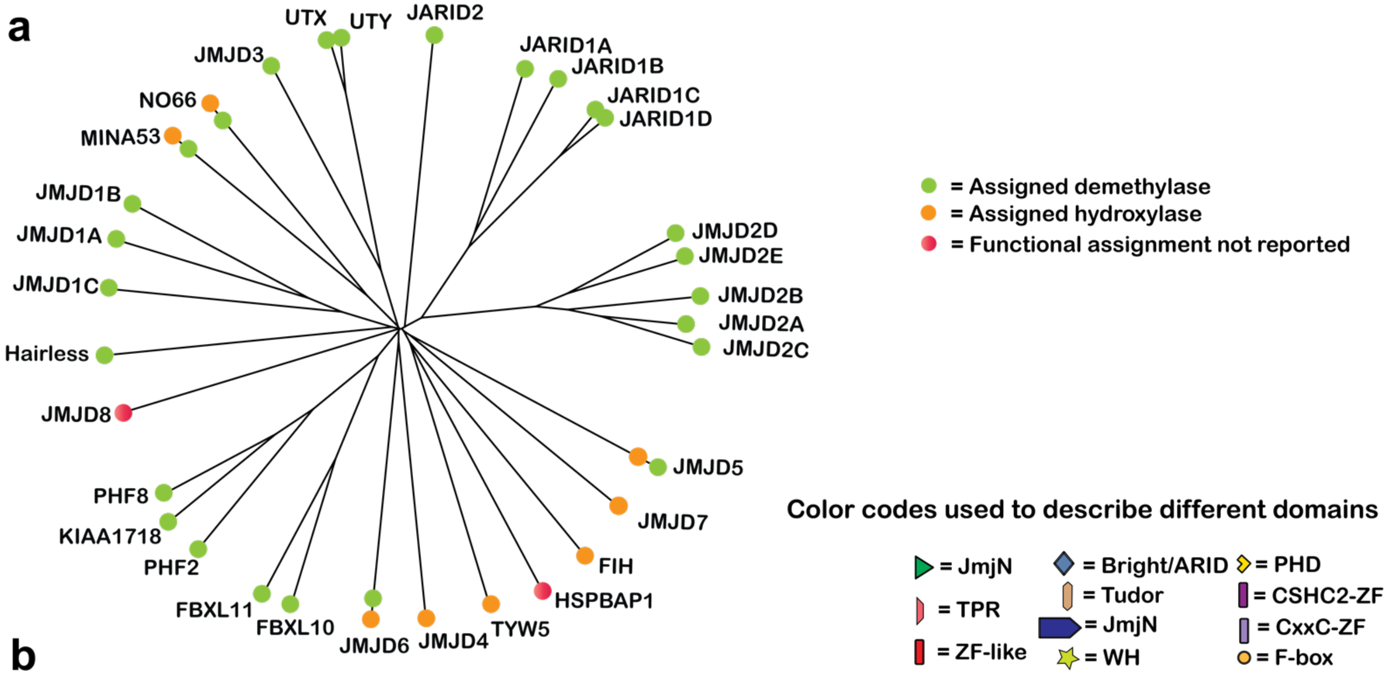

b

Gene	Domain Architecture	Protein Name	UniProtKB Accession Number
MINA53<br>NO66	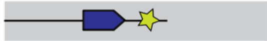	MYC induced nuclear antigen<br>Nuclear protein 66	Q8IUF8<br>Q9H6W3
JMJD3/KDM6B<br>UTX/KDM6A<br>UTY	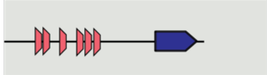	Jumonji domain containing protein 3<br>Ubiquitously transcribed tetratricopeptide repeat protein, chromosome X<br>Ubiquitously transcribed tetratricopeptide repeat protein, chromosome Y	O15054<br>O15054<br>O14607
JARID2/JMJ	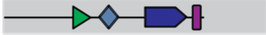	Jumonji/ARID domain containing protein 2/Protein Jumonji	Q92833
JARID1A/RBBP2/KDM5A<br>JARID1B/KDM5B<br>JARID1C/KDM5C<br>JARID1D/KDM5D	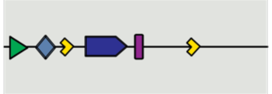	Jumonji, AT rich interactive domain 1A<br>Jumonji, AT rich interactive domain 1B<br>Jumonji, AT rich interactive domain 1C<br>Jumonji, AT rich interactive domain 1D	P29375<br>Q9UGL1<br>P41229<br>Q9BY66
JMJD2D/KDM4D<br>JMJD2E/KDM4E<br>JMJD2B/KDM4B<br>JMJD2A/KDM4A<br>JMJD2C/KDM4C<br>JMJD5	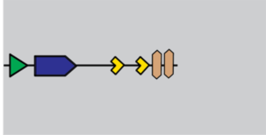	Jumonji domain containing protein 2D<br>Jumonji domain containing protein 2E<br>Jumonji domain containing protein 2B<br>Jumonji domain containing protein 2A<br>Jumonji domain containing protein 2C<br>Jumonji domain containing protein 5	Q6B0I6<br>B2RXH2<br>P29375<br>O75164<br>O94953<br>Q8N371
JMJD7<br>FIH<br>HSPBAP1/PASS1<br>TYW5<br>JMJD4<br>JMJD6	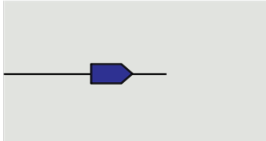	Jumonji domain containing protein 7<br>Factor Inhibiting hypoxia inducible factor 1-alpha<br>HSPB1-associated protein 1<br>tRNA wybutosine-synthesizing protein 5<br>Jumonji domain containing protein 4<br>Jumonji domain containing protein 6	P0C870<br>Q9NWT6<br>Q96EW2<br>A2RUC4<br>Q9H9V9<br>Q6NYC1
FBXL10/JHDM1B/KDM2B<br>FBXL11/JHDM1A/KDM2A	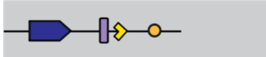	F-box and leucine-rich repeat protein 10<br>F-box and leucine-rich repeat protein 11	Q8NHM5<br>Q9Y2K7
PHF2/JHDM1E<br>KIAA1718/JHDM1D/KDM7<br>PHF8/KIAA1111	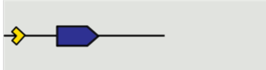	PHD finger protein 2<br>Lysine-specific demethylase 7<br>PHD finger protein 8	O75151<br>Q6ZMT4<br>Q9UPP1
JMJD8	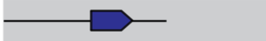	Jumonji domain containing protein 8	Q96S16
Hairless<br>JMJD1C/JHD2C<br>JMJD1A/KDM3A<br>JMJD1B/KDM3B	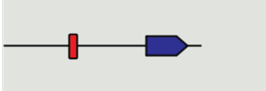	Protein hairless<br>Jumonji domain containing protein 1C<br>Jumonji domain containing protein 1A<br>Jumonji domain containing protein 1B	O43593<br>Q15652<br>Q9Y4C1<br>Q7LBC6

(a) A phylogenetic tree showing the relative positioning of the human demethylases (green circles), hydroxylases (i.e. enzymes giving stable alcohol products) (orange circles), and 2OG oxygenases

with no assigned function (red circles). The tree was constructed using Archaeptaryx v.0.9812 from ClustalW using aligned human 2OG oxygenase protein sequences[1,2]. (b) Domain architectures of the human 2OG oxygenases. ARID = AT-rich interaction domain, PHD = plant homeobox domain, TPR = tetratricopeptide repeat, WH = winged-helix, ZF = zinc finger. [The figure was adapted from Klose *et al.* 2006[3]]

Scheme 1

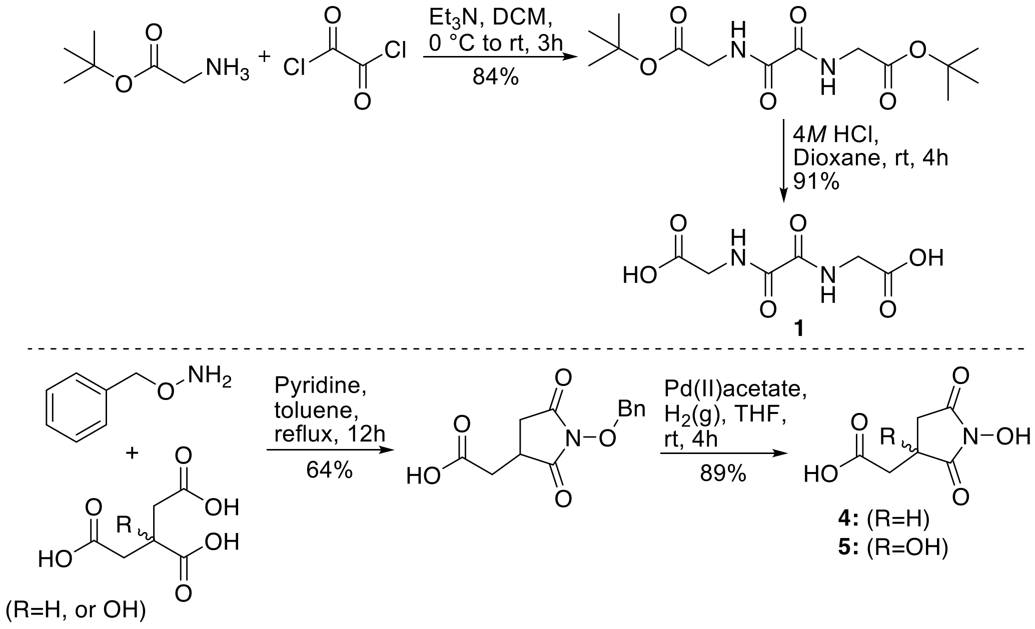

Following procedures reported previously[4,5], oxamide-N,N-diacetic acid **1** was prepared in two steps by the condensation of glycine *tert*-butyl with oxalyl dichloride, followed by hydrolysis.[4] Cyclic hydroxamic acids **4** and **5** were prepared in two steps from tricarballylic acid (R=H) and citric acid (R=OH), respectively, employing hydrogenation for deprotection[5] (Scheme 1).

di-*tert*-Butyl 2,2'-(oxalylbis(azanediyl))diacetate: (84%) white solid; **mp** 124-128 °C; $\boldsymbol{R_f}$ = 0.35 (100% $CHCl_3$); **IR**, ν_{max} (ATR)/cm^{-1}: 3325 (br), 2980 (s), 2936 (s), 1743 (s), 1671 (s), 1515 (s), 1458 (s), 1412 (s); **HRMS** (ESI) m/z calc. for $C_{14}H_{24}N_2O_6Na^+$ [M+Na] = 339.15266, found [M+Na] = 339.15239; **^{1}H NMR** (500 MHz, chloroform-*d*) δ ppm 7.90 (t, J = 5.7 Hz, 2H), 3.91 (d, J = 5.8 Hz, 4H), 1.40 (s, 18H); **^{13}C NMR** (126 MHz, chloroform-*d*) δ 167.61, 159.41, 82.45, 41.99, 27.95.

Oxamide-*N,N*-diacetic acid (1): (91%) white solid; **mp** 230 °C (dec.); $\boldsymbol{R_f}$ = 0.30 ($CHCl_3$:MeOH; 90:10); **IR**, ν_{max} (ATR)/cm^{-1}: 3297 (br), 2981 (s), 1733 (s), 1701 (s), 1653 (s), 1558 (s), 1508 (s), 1436 (s); **HRMS** (ESI) m/z calc. for $C_6H_8N_2O_6Na^+$ [M+Na] = 227.02746, found [M+Na] = 227.02767; **^{1}H NMR** (400 MHz, DMSO-*d*$_6$) δ ppm 12.75 (s, 2H), 8.97 (t, J = 6.2 Hz, 2H), 3.82 (d, J = 6.3 Hz, 4H); **^{13}C NMR** (126 MHz, DMSO-*d*$_6$) δ ppm 170.78, 160.37, 41.34.

Figure S13.

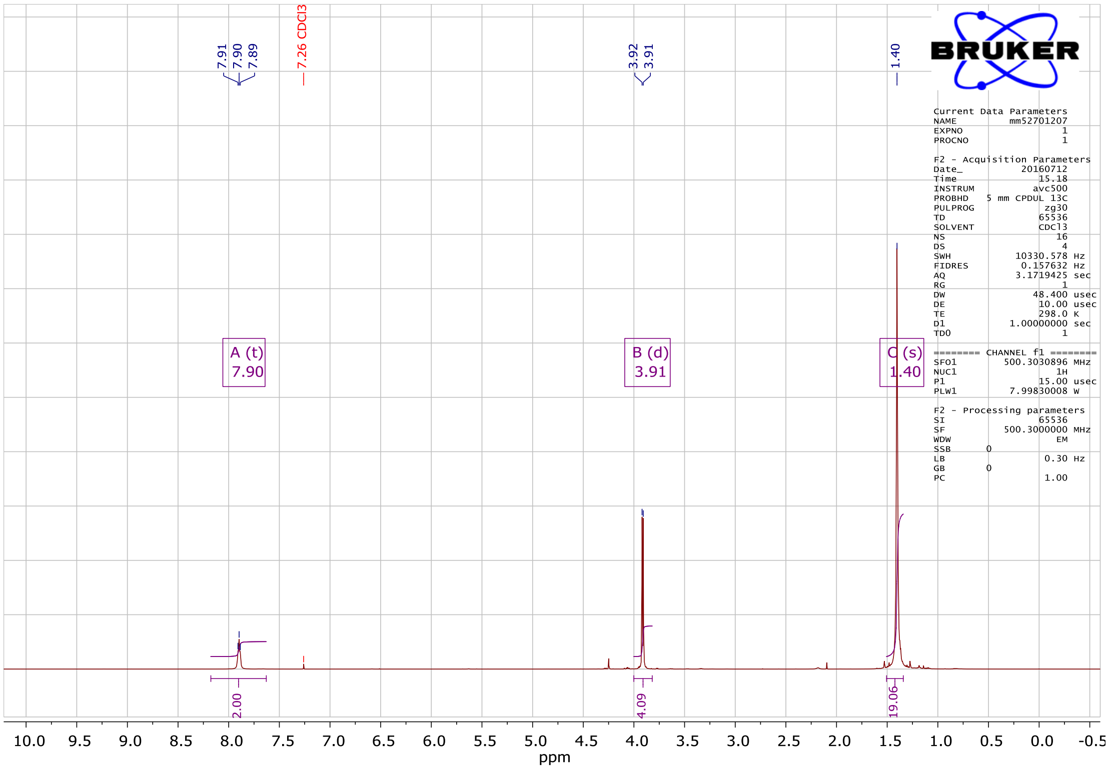

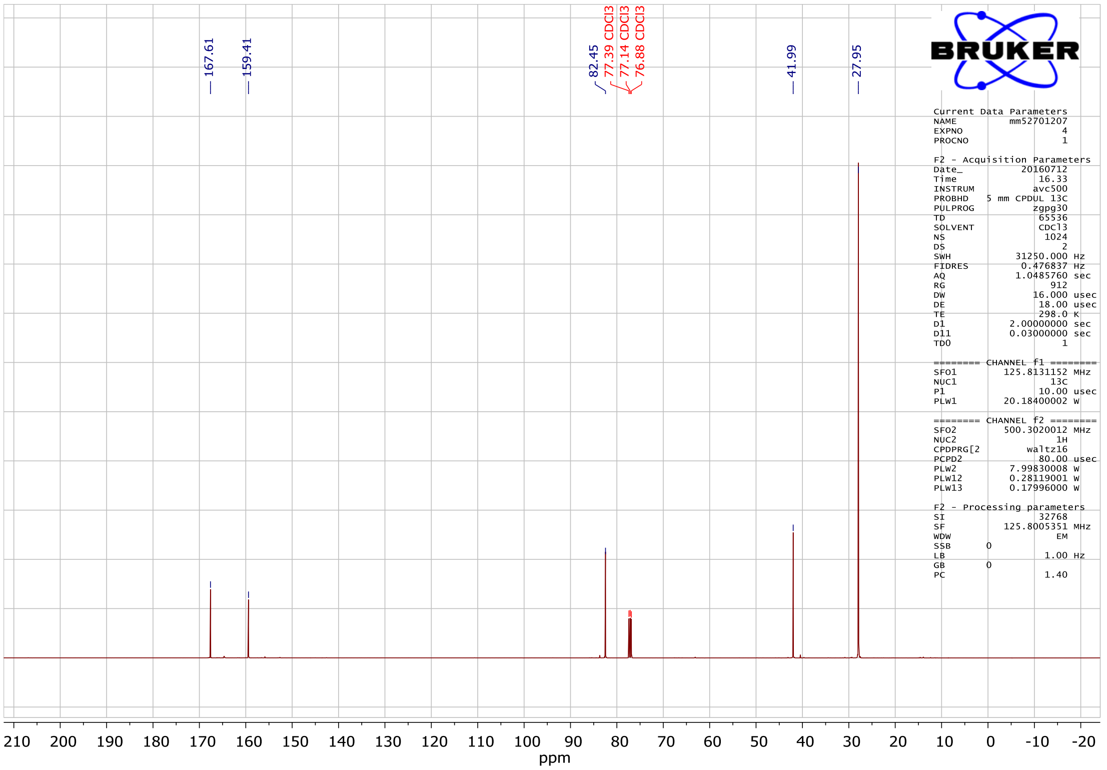

NMR spectra of di-*tert*-butyl 2,2'-(oxalylbis(azanediyl))diacetate.

Figure S14.

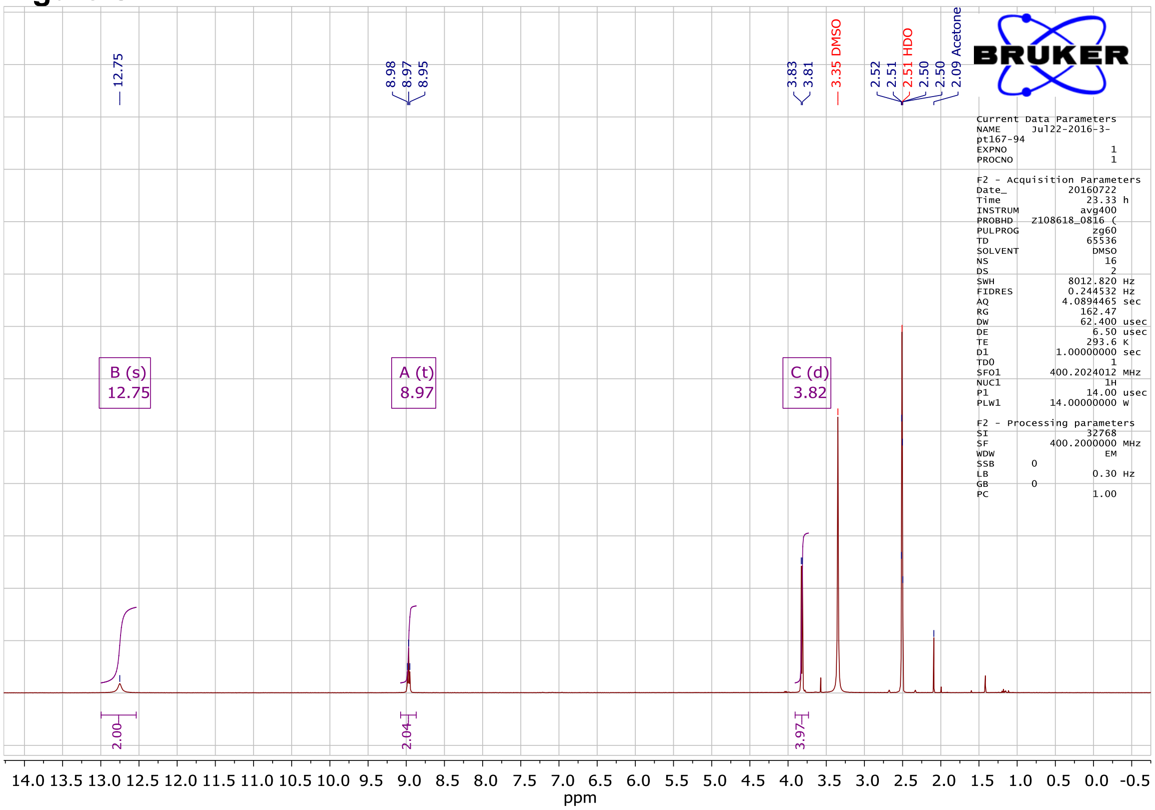

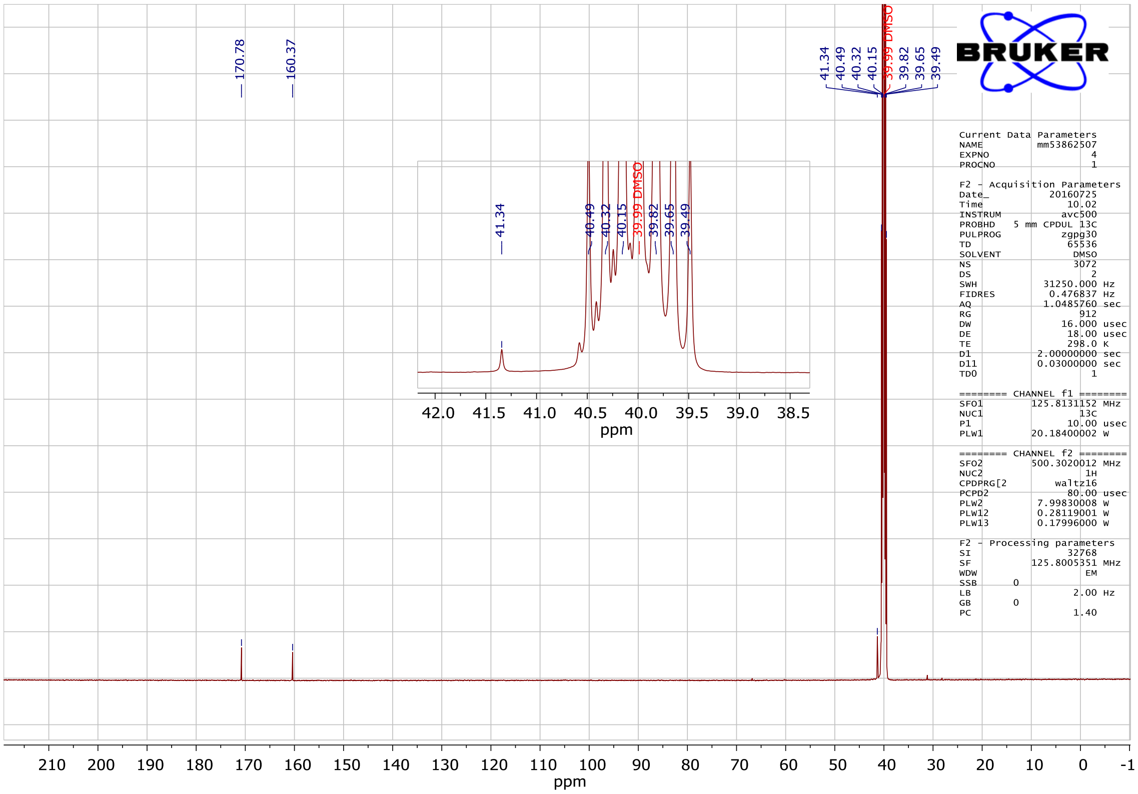

NMR spectra of oxamide-*N*,*N*-diacetic acid (1).

REFERENCES

1. Han, M. V & Zmasek, C. M. phyloXML: XML for evolutionary biology and comparative genomics. *BMC Bioinformatics* **10**, 356 (2009).
2. Larkin, M. A. *et al.* Clustal W and Clustal X version 2.0. *Bioinformatics* **23**, 2947–2948 (2007).
3. Klose, R. J., Kallin, E. M. & Zhang, Y. JmjC-domain-containing proteins and histone demethylation. *Nat. Rev. Genet.* **7**, 715–727 (2006).
4. Mecinović, J., Loenarz, C., Chowdhury, R. & Schofield, C. J. 2-Oxoglutarate analogue inhibitors of prolyl hydroxylase domain 2. *Bioorg. Med. Chem. Lett.* **19**, 6192–6195 (2009).
5. Schlemminger, I. *et al.* Analogues of dealanylalahopcin are inhibitors of human HIF prolyl hydroxylases. *Bioorg. Med. Chem. Lett.* **13**, 1451–1454 (2003).
